# Synergistic Enhancement of Mechanical and Electrochemical Properties in Grafted Polymer/Oxide Hybrid Electrolytes

**DOI:** 10.1002/smll.202404537

**Published:** 2024-08-26

**Authors:** Felix Scharf, Annalena Krude, Peter Lennartz, Moritz Clausnitzer, Gourav Shukla, Annika Buchheit, Fabian Kempe, Diddo Diddens, Pascal Glomb, Melanie M. Mitchell, Timo Danner, Andreas Heuer, Arnulf Latz, Martin Winter, Gunther Brunklaus

**Affiliations:** ^1^ Helmholtz Institute Münster Forschungszentrum Jülich GmbH IMD‐4, Corrensstraße 48 Münster Germany; ^2^ Deutsches Zentrum für Luft‐ und Raumfahrt (DLR) Helmholtz Institut Ulm (HIU) – Institut für Technische Thermodynamik Computergestützte Elektrochemie Helmholtzstraße 11 Ulm Germany; ^3^ Institut für Physikalische Chemie Universität Münster Correnstraße 28/30 Münster Germany; ^4^ MEET Battery Research Center University of Münster Corrensstraße 46 Münster Germany

**Keywords:** alumina, dry electrolyte, grafted oxide particles, hybrid electrolyte, lithium metal, poly(caprolactone), solid electrolyte, solid‐state batteries

## Abstract

Lithium metal batteries operated with high voltage cathodes are predestined for the realization of high energy storage systems, where solid polymer electrolytes offer a possibility to improve battery safety. Al_2_O_3__PCL is introduced as promising hybrid electrolyte made from polycaprolactone (PCL) and Al_2_O_3_ nanoparticles that can be prepared in a one‐pot synthesis as a random mixture of linear PCL and PCL‐grafted Al_2_O_3_. Upon grafting, synergistic effects of mechanical stability and ionic conductivity are achieved. Due to the mechanical stability, manufacture of PCL‐based membranes with a thickness of 50 µm is feasible, yielding an ionic conductivity of 5·10^−5^ S cm^−1^ at 60 °C. The membrane exhibits an impressive performance of Li deposition in symmetric Li||Li cells, operating for 1200 h at a constant and low overvoltage of 54 mV and a current density of 0.2 mA cm^−2^. NMC_622 _| Al_2_O_3__PCL | Li cells are cycled at rates of up to 1 C, achieving 140 cycles at >80% state of health. The straightforward synthesis and opportunity of upscaling as well as solvent‐free polymerization render the Al_2_O_3__PCL hybrid material as rather safe, potentially sustainable and affordable alternative to conventional polymer‐based electrolytes.

## Graphical abstract

This work introduces a new kind of solid and fully dry hybrid electrolyte. Sustainable Polycaprolactone, grafted on commercial and affordable Al_2_O_3_ nanoparticle exhibits synergistic effects. Covalent bonds between polymer and oxide ceramics lead to improved ionic conductivity and enhanced mechanical properties of a solid electrolyte suitable for lithium metal batteries.

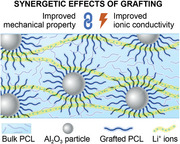

## 1 Introduction

All‐solid‐state batteries that operate without volatile flowable components might improve the overall operational safety of secondary batteries, potentially enabling the application of higher capacity anodes such as silicon or lithium metal.^[^ ^1^, ^2^, ^3^ ^]^ However, both promising anodes may suffer from substantial volume changes upon cycling, that solid electrolyte separators are required to sufficiently withstand or even counteract electrochemical performance losses or cell capacity fading imposed by mechanical strain, including issues of limited reversibility of Li inventory arising from eventually inhomogeneous lithium deposition.^[^ ^4^ ^]^ In this respect, polymer electrolytes and polymer artificial solid electrolyte interphases might constitute versatile options, since their adjustable mechanical and adhesive properties facilitate reasonable contacts at electrode interfaces even at conditions of lower pressure operation of solid‐state batteries.^[^ ^5^ ^]^ In recent years, many polymer systems were explored to possibly go beyond the established poly(ethylene oxide), including poly(nitriles), carbonyl‐coordinating polymers, and hydrogen‐bonding materials derived from poly(alcohols) or poly‐(amines), respectively, as well as poly(caprolactones).^[^ ^6^, ^7^ ^]^ Nevertheless, efforts are still required to boost fast‐charge capability of currently available polymer electrolytes and compatibility with higher voltage cathodes without limiting mechanical stability of the respective polymer membranes. Various strategies were developed to establish compromises among the conflicting demands of ionic conductivity and mechanical properties, where a rather promising approach includes manufacture of polymer blends or tailored copolymers.^[^ ^8^ ^]^ Other strategies invoke cross‐linking of polymer constituents, addition of ceramic fillers, or infiltration into (often polymer‐based) support matrices.^[^ ^6^, ^9^, ^10^, ^11^ ^]^ In contrast, ceramic electrolytes may combine superior mechanical strength with sufficiently good ionic conductivity, though their low ductility renders processing challenging while their rigid surfaces could lead to insufficient interfacial contacts and thus high cell impedance.^[^ ^12^ ^]^ Notably, balancing of ionic conductivity, mechanical strength, adhesive properties and ductility is feasible when designing so‐called hybrid electrolytes. Thus, in the present work, we focus on ceramic‐in‐polymer hybrid electrolytes, exploiting ceramic particles for a direct grafting of polymers, in this way yielding polymer brushes that afford properties quite similar to star‐like polymers, depending on the brush length, ceramic particle sizes, size distribution and available surface functionalities, respectively.^[^ ^13^, ^14^ ^]^ Here, ceramic particles could either afford intrinsic ionic conductivity or merely be exploited as inert fillers. Available compounds such as lithium lanthanum zirconium oxide (LLZO) or lithium aluminum titanium phosphate (LATP) might boost the overall ionic conductivity upon polymer grafting, but eventually suffer from surface reactivity or instability against lithium metal electrodes and the abundant presence of structural lithium that does not contribute to the charge carrier transport.^[^ ^15^ ^]^ Inert ceramics including Al_2_O_3,_ TiO_2_ or SiO_2_ are readily available as commercially produced nanomaterials and were employed as inorganic fillers in polymer electrolytes, adjusting the viscoelastic properties of the hybrid electrolytes.^[^ ^9^, ^16^, ^17^ ^]^ In case of semi‐crystalline polymers such as PEO, poly(vinylidene fluoride) (PVdF) or poly(acrylonitrile) (PAN), an addition of inorganic fillers mechanically may soften the electrolytes, hence limiting the actual degree of crystallinity.^[^ ^18^, ^19^ ^]^ This also applies to semi‐crystalline poly(caprolactone) (PCL), which was blended with nano‐fillers such as hexagonal boron nitride (h‐BN), TiO_2_ or Al_2_O_3_, respectively.^[^ ^20^, ^21^ ^]^ Notably, the ionic conductivity of PCL increases to 1·10^−4^ S cm^−1^ at 60 °C upon addition of Al_2_O_3_ compared to pristine PCL polymer electrolytes (8·10^−5^ S cm^−1^ at 60 °C). Also, PCL‐based systems afford higher lithium transference numbers that contribute to an overall better ionic conductivity than reported for PEO‐based materials, accompanied by limited polarization effects of the cells.^[^ ^6^ ^]^ Since in theory, anion drift velocities are associated with lithium dendrite propagation, a lower anion mobility might be beneficial for more homogeneous lithium deposition and better reversibility of Li inventory.^[^ ^22^ ^]^ However, PCL‐based polymers may encounter mechanical instability at elevated temperatures, owing to their lower melting point compared to PEO‐based separators.^[^ ^8^ ^]^ To overcome this challenge, while accounting for a scalability of the synthetic approach, a hybrid polymer made from Al_2_O_3_ particles grafted with PCL polymer is introduced. Due to covalent bonds among polymer moieties and particle surface hydroxyl groups, 3D networks with enhanced mechanical properties are obtained, providing better compatibility of the constituents while avoiding macro‐phase separation and nanoparticle agglomeration.^[^ ^23^, ^24^ ^]^ Motivated by reports of PEO/PEG grafted onto either SiO_2_ or Al_2_O_3_ particles, where highly increased ionic conductivities were presented^[^ ^23^, ^24^, ^25^, ^26^ ^]^ and expanding on our recently introduced beyond‐PEO hybrid concept,^[^ ^10^ ^]^ the suitability of PCL‐grafted ceramic particles as all‐dry solid polymer hybrid electrolyte in lithium metal‐based batteries (LMBs) is explored. To the best of our knowledge, this approach is reported for the first time. Notably, the presented experimental and simulated data are intended as proof‐of‐concept that provides original insights and opportunities for the design of a new type of PCL‐based hybrid electrolytes.

## 2 Results and Discussion

**Figure 1:**
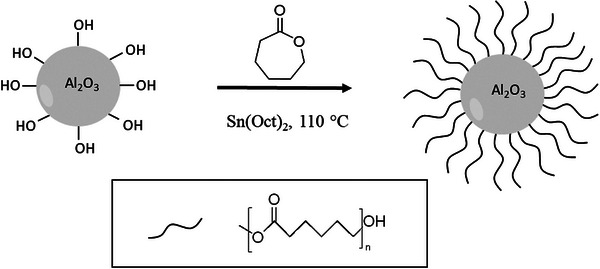
Schematic representation of the grafting process and chain growth of ε‐caprolactone onto Al_2_O_3_ nano‐particles by surface‐initiated ring‐opening polymerization, exploiting present hydroxyl moieties. Note that the compound Sn(Oct)_2_ represents tin(II) 2‐ethylhexanoate, the catalyst invoked for the ring‐opening polymerization.

In an attempt to establish an all‐dry beyond‐PEO materials design concept, the hybrid polymer electrolyte Al_2_O_3__PCL (random mixture of polymer grafted particle and linear bulk polymer, with 20 wt.% of Al_2_O_3_) is introduced, that is made by grafting of poly(caprolactone) (PCL) onto Al_2_O_3_ nano‐particles (**Figure** **1**). The ratio of 20 wt.% showed the best performance in terms of conductivity and mechanical properties (see in the Supporting Information). The reaction comprises straightforward surface‐initiated ring‐opening polymerization where both grafted polymer and linear PCL polymer are produced.^[^ ^27^ ^]^ From size exclusion chromatography (SEC) analysis, a molar mass M_w_ of 40 kg mol^−1^ was experimentally determined for linear PCL, which is formed concurrently with grafted polymer (Figure S3, Supporting Information). It is assumed that in view of the reaction conditions, the grafted PCL chains will likely reflect a similar molar mass distribution as the linear PCL, in agreement with rheological data (*vide supra*, see below). Since Al_2_O_3_ nano‐particles are readily dispersed within the liquid monomer ε‐caprolactone, no additional solvents are required for the reaction, hence avoiding subsequent removal of residual and potentially harmful solvents.^[^ ^6^ ^]^

### 2.1 Synthesis and Thermal Characterization

**Figure 2:**
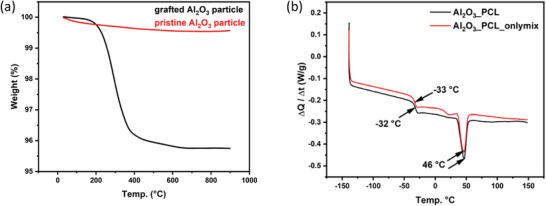
a) TGA curves of PCL‐Al_2_O_3_ and pristine Al_2_O_3_ particles and b) DSC curves of Al_2_O_3__PCL and Al_2_O_3__PCL_onlymix electrolyte membranes.

Notably, the Al_2_O_3__PCL synthesis reflects a straightforward one‐pot reaction where no complex or additional intermediate steps are required for grafting of the particles, refraining from surface functionalization based on for example silyl ethers. In view of potential application in batteries, sufficient availability and scale‐up of the electrolyte materials are highly important. Up to 500 g of Al_2_O_3__PCL were successfully produced in a 2 L reactor (Figure S13, Supporting Information), and even at laboratory scale, the precursor costs for Al_2_O_3__PCL manufacture are rather affordable when comparing the starting materials ɛ‐caprolactone and Al_2_O_3_ nano‐particles with commercially available PEO variants. Since poly(caprolactone) is potentially biodegradable, the hybrid electrolytes Al_2_O_3__PCL could in principle be considered as sustainable alternative to conventional liquid electrolytes.^[^ ^28^ ^]^ Thermogravimetric analysis (TGA) was performed to probe successful grafting of PCL onto Al_2_O_3_ nano‐particles, comparing mass losses in the temperature range (25–900 °C) of both the pristine Al_2_O_3_ nano‐particles and PCL grafted Al_2_O_3_ particles (**Figure** **2**a). Note that the PCL grafted Al_2_O_3_ particles were separated from bulk linear PCL polymer by washing with THF and subsequent ultrasonic treatment. Pristine Al_2_O_3_ nano‐particles exhibit a small and uniform weight loss of 0.5 wt.%, whereas in case of PCL‐grafted Al_2_O_3_, a weight loss of 4 wt.% occurs in the temperature range of (200–400 °C), attributed to covalently bonded PCL.^[^ ^27^ ^]^ For comparison, TGA data of pristine PCL was also obtained, where the mass loss occurs in a similar temperature range compared to PCL‐grafted Al_2_O_3_ (Figure S5, Supporting Information).

**Figure 3:**
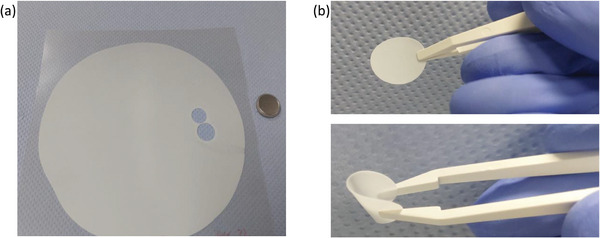
a) Example of a 1.2 g Al_2_O_3__PCL membrane produced by hot‐pressing, and b) mechanical handling of the corresponding Al_2_O_3__PCl discs for cell assembly.

The corresponding hybrid polymer membranes were manufactured by a solvent‐free approach, though production from solution casting is feasible. Notably, dry melt processing avoids evaporation of residual solvents within the membrane, that may result in side reactions or misinterpretation of electrochemical data. Upon preparation of the PCL‐based polymer electrolytes, the conducting salt LiTFSI was added at a [C = O]/[Li] ratio of 5:1, reflecting a Li ion concentration of 1.752 mol kg^−1^. It should be emphasized that reasonably thin Al_2_O_3__PCL membranes (**Figure** **3**) with an average thickness of 50 µm were fabricated, which is considered as favorable in view of higher energy densities to be achieved in case of Al_2_O_3__PCL‐based NMC||Li cells.^[^ ^29^ ^]^

Upon exploiting extrusion and heated roll pressing, it may be possible to produce larger quantities of Al_2_O_3__PCL membranes continuously, thereby further thinning the Al_2_O_3__PCL membranes, which, however, is beyond the scope of the current work. Differential Scanning Calorimetry (DSC) measurements were performed to analyze the impact of PCL‐grafting on the resulting physical properties of the polymer electrolyte, yielding both the glass transition temperature (*T_g_*) and melting point (*T_m_*) (Figure 2b). Al_2_O_3__PCL was compared with blends of linear PCL and pristine Al_2_O_3_ nano‐particles (denoted as Al_2_O_3__PCL_onlymix); the respective mass fractions of inorganic particles and salt were the same in both cases. The chain length of linear PCL polymer corresponds to the chain length of bulk linear PCL (45.000 g mol^−1^) resulting from Al_2_O_3__PCL synthesis. Note that the particle size of the Al_2_O_3_ was 300 nm. The corresponding *T_g_* values of Al_2_O_3__PCL and Al_2_O_3__PCL_onlymix amount to −32 and −33 °C, respectively, while the *T_m_* in both cases is 46 °C. Also, the electrolyte membrane heat capacities were determined, yielding a higher specific heat capacity for the electrolyte membranes made from Al_2_O_3__PCL (1.0152 kJ kg^−1^ K^−1^) compared to Al_2_O_3__PCL_onlymix (0.8797 kJ kg^−1^ K^−1^). Note that the heat capacities were derived below the *T_g_* of the two materials. An increase in specific heat capacity can be noted based on the integral of heat over temperature (a curve shift in Y direction),^[^ ^30^ ^]^ reasonably indicating that the mobility within the PCL‐grafted material is more limited with respect to plain mixtures of PCL‐polymer and Al_2_O_3_ nano‐particles.^[^ ^30^ ^]^ The second heating ramp during the DSC measurement (see Figure S6, Supporting Information) demonstrates that Al_2_O_3__PCL no longer exhibits a melting point, whereas a minor peak is visible in case of Al_2_O_3__PCL_onlymix, clearly indicative of minor fractions of crystalline structures present in the samples.

### 2.2 Mechanical Characterization

**Figure 4:**
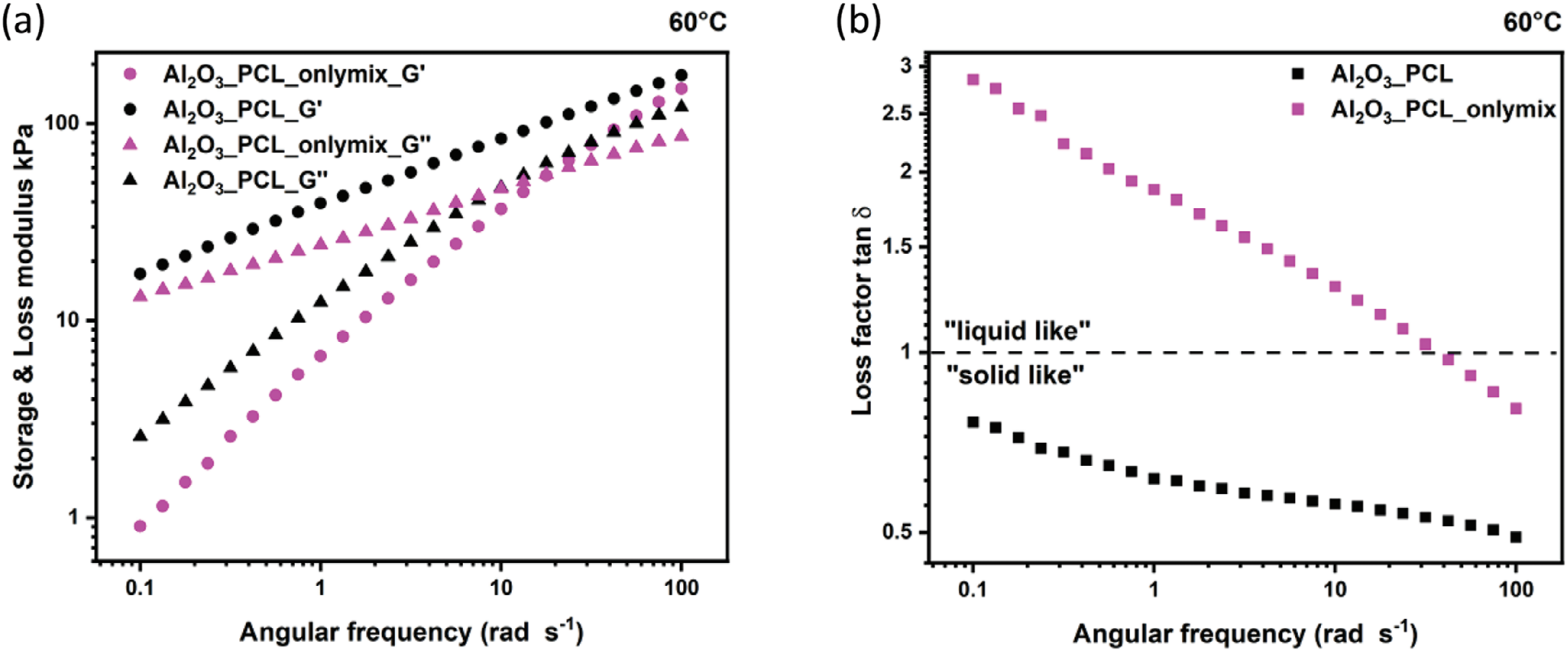
a) Storage (G´) and loss moduli (G´´) as well as b) loss factor tan δ versus angular frequency in case of Al_2_O_3__PCL and Al_2_O_3__PCL_onlymix electrolyte membranes at 60 °C.

Upon grafting, a formation of covalent bonds between PCL and Al_2_O_3_ nano‐particles occurs, in this way changing the mechanical features of the electrolyte membranes, as evidenced based on the corresponding storage and loss moduli determined from the rheological measurements. In particular, the moduli of Al_2_O_3__PCL were compared to a blend of linear PCL and Al_2_O_3_ nano‐particles (Al_2_O_3__PCL_onlymix) (**Figure** **4**a) at a temperature of 60 °C. Indeed, the storage modulus G' and loss modulus G″ of viscoelastic compounds provide information on the amount of energy that can be stored in the elastic fraction, while the energy dissipated as heat reflects the viscous fraction. If a storage modulus exceeds the loss modulus, then the material is considered as predominantly elastic, which is the case for Al_2_O_3__PCL, whereas the blend appears more as a viscous fluid.^[^ ^31^ ^]^ This is also illustrated by the loss factor tan δ (Figure 4b), which is the ratio of loss and storage modulus (tan δ  =  *G*′′/*G*′). Though the Al_2_O_3__PCL is completely amorphous at 60 °C, as the *T*_m_ is 46 °C, it has the properties of a solid.

In agreement with previous reports, the storage modulus G′ tends to increase upon addition of polymer‐grafted nano‐particles, provided that the respective polymer matrix molecular weight is comparable to the molecular weight of the grafted polymers.^[^ ^32^ ^]^ Due to the invoked *in situ* polymerization process, this condition can be considered as fulfilled for the introduced hybrid polymer electrolytes, in agreement with available rheological data. Increasing the mechanical stability of polymer electrolytes is not only advantageous for producing thin membranes, but is also helpful in suppressing or limiting lithium dendrites.^[^ ^33^ ^]^ Due to the higher storage modulus of the grafted system, higher pressures can be applied to the hybrid polymer electrolyte upon cell operation without damaging it. In addition to improved lithium dendrite suppression, this can also result in a reduction of charge transfer resistances.^[^ ^34^ ^]^ In comparison to PCL‐based polymer electrolytes known from the literature and the Al_2_O_3__PCL_onlymix, the mechanical properties of the hybrid polymer electrolyte Al_2_O_3__PCL could be notably improved by grafting.

### 2.3 Charge Transport Properties

**Figure 5:**
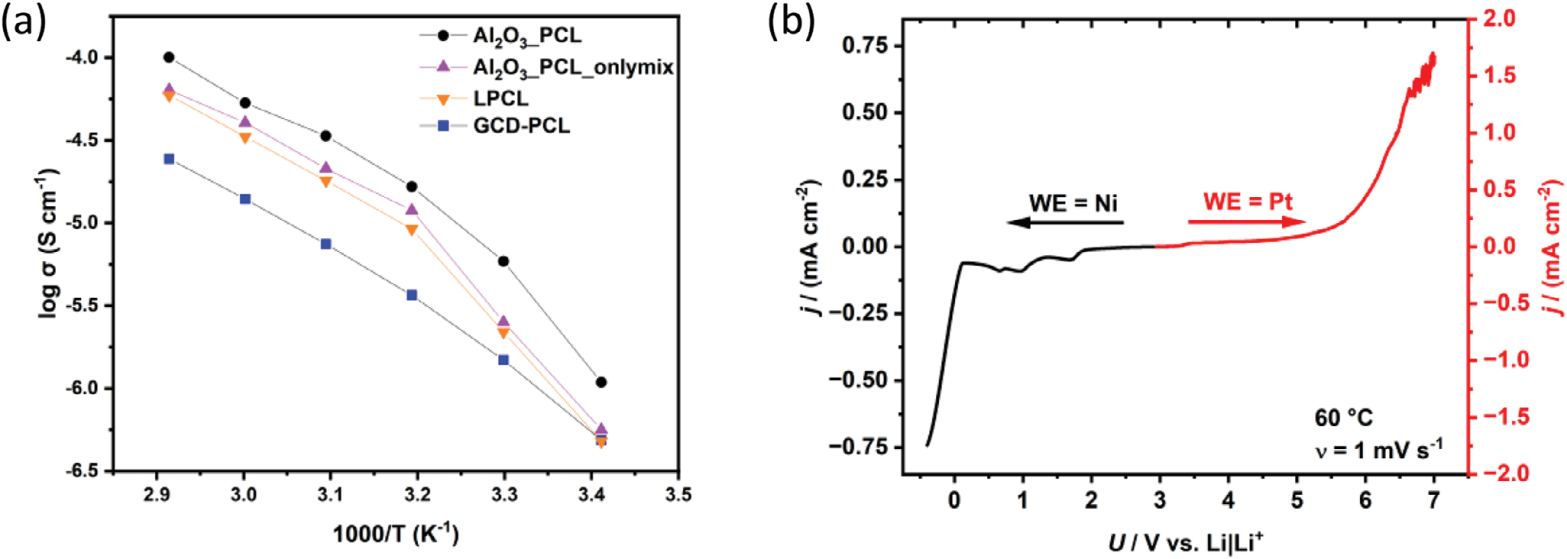
a) Temperature‐dependent overall ionic conductivities of Al_2_O_3__PCL, Al_2_O_3__PCL_onlymix, LPCL and GCD‐PCL, respectively, and b) the LSV curve of Al_2_O_3__PCL at a scan rate of 1 mV s^−1^.

Previously reported PCL‐based electrolytes achieved the highest ionic conductivity at a ratio of 5:1 [C = O]/[Li], with Al_2_O_3__PCL affording an ionic conductivity of 0.053 mS cm^−1^ at 60 °C at this concentration.^[^ ^6^, ^35^ ^]^ It is apparent that in case of Al_2_O_3__PCL, the mechanical properties of poly(caprolactone) were considerably boosted without overly compromising the achievable ionic conductivity, as shown by electrochemical impedance spectroscopy data (**Figure** **5**a). Linear PCL (LPCL) was compared to both a mixture of Al_2_O_3_ particles and LPCL (Al_2_O_3__PCL_onlymix) and Al_2_O_3__PCL, as well as to a PCL‐grafted electrolyte from previous work (cyclodextrin ring grafted with poly(caprolactone): GCD‐PCL).^[^ ^10^ ^]^ Note that compared to GCD‐PCL (0.014 mS cm^−1^ at 60 °C) the resulting ionic conductivity was notably higher, highlighting the benefits of grafting onto ceramic particles, whereas the ionic conductivities of LPCL (0.033 mS cm^−1^ at 60 °C) and Al_2_O_3__PCL_onlymix (0.04 mS cm^−1^ at 60 °C) are comparable. A comparison with other PCL‐based electrolyte systems also shows an improvement in ionic conductivity (Table S4, Supporting Information).^[^ ^6^, ^10^ ^]^ For the three systems Al_2_O_3__PCL, Al_2_O_3__PCL_onlymix and LPCL, a change in the slope of the curve between 30 and 40 °C can be observed (Figure 5a). This observation correlates with the DSC data, where the polymers started a transformation from crystalline to amorphous phases already at 30 °C (Figure 2b).

Note that the slope of the curve (see Figure 5a) represents an apparent activation energy required for ion transport within the polymer electrolytes; above 30 °C the required activation energy is lower due to the higher mobility of the polymer chains (E_a_ ≈52 kJ mol^−1^ for Al_2_O_3__PCL). However, in both cases, below and above the melting point, the ionic conductivity follows an Arrhenius‐type behavior (that is, a linear dependence of ionic conductivity with temperature). In case of Al_2_O_3__PCL_onlymix and LPCL, the change in activation energy was even more pronounced than observed for the hybrid electrolyte Al_2_O_3__PCL, which most likely indicates the presence of a higher proportion of amorphous structures within Al_2_O_3__PCL.^[^ ^36^, ^37^ ^]^ In addition to ionic conductivity, the lithium transference number of Al_2_O_3__PCL was determined, yielding t^+^ = 0.65, which is similar to other PCL‐based electrolytes.^[^ ^38^ ^]^ In contrast, transference numbers of PEO‐based electrolytes are around t^+^ = (0.1–0.3).^[^ ^39^ ^]^ A linear sweep voltammetry measurement was performed to determine the electrochemical stability window (ESW), revealing an ESW of ≈5 V, as determined by a cut‐off current density of 0.1 mA cm^−2^ (Figure 5b).

### 2.4 MD Simulation

**Figure 6:**
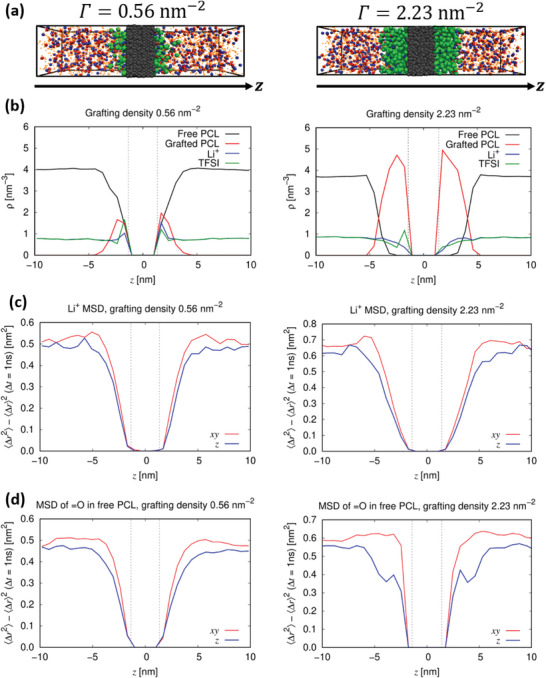
a) Snapshots of the simulated systems with different grafting densities (0.56 and 2.23 PCL chains per nm^2^; gray: Al_2_O_3_, red: Li^+^, blue: TFSI, green: grafted PCL, orange dots: free PCL). b) Corresponding number densities of grafted and non‐grafted PCL chains as well as cations and anions as a function of the distance z to the center of the Al_2_O_3_ slab. Mean squared displacements (MSDs) of c) the lithium ions and d) an average double bonded oxygen atom in a PCL chain as a function of z (coordinate perpendicular to the PCL/Al_2_O_3_ interface) in parallel (x,y) and perpendicular direction (z) to the interface. The dashed vertical lines in (c) and (d) indicate the position of the Al_2_O_3_ interface.

Molecular dynamics (MD) simulations were performed for the concentrated polymer brush region of PCL‐grafted Al_2_O_3_ nanoparticles. The simulation was carried out for a simulation box with a size of ≈5.78 × 4.96 × 20 nm^3^ and a 3‐nm‐thick slab of filler material (Al_2_O_3_) in the center with polymer chains made from five repeating units that have been grafted onto the nanoparticle surfaces (**Figure** **6**). Two grafting densities Γ, with 16 and 64 grafted PCL chains on the total surface area of 28.67 nm^2^, were considered, corresponding to number densities of Γ = 0.56 and 2.23 PCL chains per nm^2^ of Al_2_O_3_ surface area, respectively.

Figure 6a shows two representative snapshots of the simulated polymer systems, representing two different grafting densities (left: 0.56 PCL chains per nm^2^; right: 2.23 PCL chains per nm^2^). It is apparent that for the smaller grafting density, the grafted chains are rather coiled as compared to the system with the higher grafting density, in which the chains adapt rather stretched conformations. Figure 6b displays corresponding density profiles for grafted and non‐grafted PCL monomers for different positions along the *z*‐coordinate normal to the Al_2_O_3_ surface, in addition to the respective charge carrier density profiles (Li^+^ and TFSI). It is observed that while for the system with Γ = 0.56 nm^−2^ some of the non‐grafted PCL chains are in direct contact with the Al_2_O_3_ surface, the corresponding density decays to zero within the grafted region for the system with the higher grafting density of 2.23 nm^−2^. This is in line with de Gennes theory, predicting that for sufficiently high grafting densities the non‐grafted chains are expelled from the grafted domains due to entropic reasons.^[^ ^40^ ^]^ However, it should be mentioned that the PCL chains subjected to the MD simulations are too short to fully reach the expected scaling regime, although other MD studies using PEO support de Gennes theory.^[^ ^41^ ^]^ Notably, according to theory, a linear scaling of the chain extension with the number of monomers is predicted for grafted chains in case of sufficiently high grafting densities, whereas a square‐root scaling is anticipated in case of lower grafting densities and for non‐grafted chains.^[^ ^40^ ^]^

From the charge carrier density distribution, a certain layering near the Al_2_O_3_ interface due to the dipole arising from the surface termination is noticed (Figure 6b), similar to other recent MD studies of an interface between LLZO and (non‐grafted) PEO.^[^ ^42^, ^43^, ^44^, ^45^ ^]^ Since in the considered simulation setup the Al_2_O_3_ slab was obtained by cutting a periodic Al_2_O_3_ supercell, the resulting structure of both surfaces is not identical. This has an impact on the accumulation of charge carriers near the surface in the domain of the grafted PCL chains, as in one case the Li^+^ ion peak directly at the interface is higher, whereas in the other case the TFSI peak is larger.

Figure 6c exhibits the mean squared displacement (MSD) parallel and perpendicular to the Al_2_O_3_ surface as a function of position, evaluated on a time scale of 1 ns. The Li^+^ mobility is slowest close to the surface of the Al_2_O_3_ particles by up to one order of magnitude as compared to regions that are 4–5 nm away from the particle surface. This slowdown is present for both grafting densities, and can likely be attributed to both direct interactions between Li^+^ and the Al_2_O_3_ surface as well as to the lower polymer chain mobility due to anchoring/grafting. Another remarkable feature is that the MSDs for both grafting densities are anisotropic, where the lithium ion mobility parallel to the Al_2_O_3_ surface is higher than in the perpendicular direction (Figure 6c). Moreover, this effect is slightly more pronounced for the higher grafting density. While in the immediate vicinity of the surface such behavior might be expected, Figure 6c demonstrates that the anisotropically enhanced diffusion extends even beyond the region of the PCL brush into the domain of free PCL chains, until the isotropic bulk behavior is recovered for large distances to the surface. Although the observed anisotropy could partly be related to slight structural ordering effects at the boundary between the grafted and the free PCL chains (see Figure S2, Supporting Information), a certain degree of dynamical anisotropy persists even beyond z = ± 5 nm, for which structural isotropy of the bulk is recovered (Figure S2, Supporting Information). A similar dynamical anisotropic behavior is observed for the MSD of free PCL chains (Figure 6d). Notably, these findings are in line with the density‐dependent lubrication properties of grafted polymer brushes in contact with a polymer melt,^[^ ^46^ ^]^ although it should be stressed that the latter mechanism was observed under shear.

In total, one might therefore assume that the experimentally observed increase of the ionic conductivity results from an enhanced segmental mobility of free PCL chains in the vicinity of the grafted PCL brushes. This is also in line with the observation that grafting PCL on a *molecular* species, such as in GCD‐PCL, rather than an oxidic surface does not result in improved transport properties (Figure 5a). Importantly, the local enhancement depends on the actual PCL‐grafting density due to the fact that the dynamical increase within the free PCL/grafted PCL boundary region is larger for the higher grafting density. Here, further optimization of this control parameter seems to be a viable strategy for future materials design. Moreover, preliminary simulations of PCL grafted on an LLZO surface indicate that the dynamical enhancement could be even larger than for Al_2_O_3_. It should be stressed that additional enhancement mechanisms of the polymer‐based ion transport could be induced by longer grafted chains employed experimentally, which will not be observed from the present MD simulations using short PCL chains. Due to the structural ordering predicted by de Gennes theory^[^ ^40^ ^]^ for sufficiently high grafting densities, the effective radius of the nanoparticles would increase significantly in this limit. In this way, an additional potentially modified lithium ion transport mechanism within the grafted domain could even extend over several hundred nanometers.

### 2.5 Conductivity Simulation

To develop a better fundamental understanding how such an enhancement of the local ion transport mechanisms affects the conduction properties on larger scales, additional continuum simulations of the effective conductivity of the introduced hybrid electrolytes were also performed, where the data served for inverse analysis.

**Figure 7:**
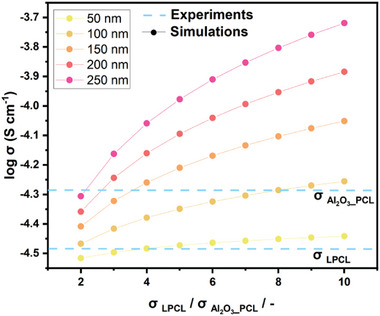
Impact of the simulated layer thickness of grafted polymer on the effective conductivity of Al_2_O_3__PCL hybrid electrolytes and comparison with experimental data (dashed lines).

**Figure** **7** displays the simulated effective conductivities of the considered hybrid electrolytes Al_2_O_3__PCL, assuming the presence of a homogeneous layer with enhanced ionic conductivity around the Al_2_O_3_ nano‐particles originating from grafting of the polymer chains (brush). For the simulations, the thickness of the layer with enhanced conductivity is varied as is the ionic conductivity relative to bulk PCL polymer. A comparison of simulation data and experimental results obtained for the hybrid polymer electrolytes provides insights into underlying effects that are likely responsible for the noticeable increase in the overall ionic conductivity. Note that the corresponding thickness of the grafted polymer layer can be estimated based on scaling methods from polymer theory,^[^ ^40^, ^47^ ^]^ whereas experimental data from size exclusion chromatography (SEC) analysis revealed a molar mass M_w_ of 40 kg mol^−1^ for bulk PCL, whose chains could extent 20 to 250 nm, depending on the actual grafting density. However, certain key parameters, such as the actual grafting density, are experimentally not readily accessible. Based on preliminary estimates and including observations of MD simulations above, a layer thickness of up to a few hundred nanometers in case of the stretched PCL chains appears very plausible. Thus, in simulations, this thickness is varied from 50 to 250 nm, respectively. At a thickness of 100 nm, a percolating network of the conductive polymer layers starts to emerge, in this way providing pathways for faster ionic conduction throughout the micro‐structured polymer hybrid electrolytes.

Upon comparison of the simulated ionic conductivities with the experimentally derived values, the plausibility of the exploited simulation parameters is assessed, also corroborating classification of the observed ionic conductivity enhancements. At a layer thickness of 50 nm, even a rather high increase of ionic conductivity within grafted polymer layers results in a minor overall enhancement of the effective conductivity, though with increasing layer thickness, the corresponding volume fraction of the highly conductive polymer rises notably in microstructure. At a thickness of 100 nm, the experimentally measured conductivity of the hybrid electrolytes is reached with an approximately eightfold increase in ionic conductivity relative to bulk PCL polymer. At layer thicknesses ranging between 150 and 250 nm, the boost of ionic conductivity requires a factor of 2 up to 4. The MD simulations indicate that a geometrical stretching of the PCL polymer chains, facilitating Li ion transport, may account for the substantial increase in the effective ionic conductivity within the considered Al_2_O_3__PCL hybrid electrolytes. In theory, an even more notable increase in effective ionic conductivity is anticipated for polymers with longer grafted chains (that is, polymers with a higher number of monomers, larger monomer size and optimized grafting density)^[^ ^40^ ^]^ and overall better charge carrier transport. This may include a template effect in the outer semi‐dilute brush region where grafted polymer chains are interpenetrated by free (linear) polymer chains, effectively increasing the size of the grafting layer. As the MD simulation demonstrated, interfacial effects do not contribute to the observable higher ionic conductivity at the regions near the Al_2_O_3_/grafted PCL interface. Considering the experimental data, a grafted molecular weight of 40 kg mol^−1^ would yield an up to 250 nm large grafting layer where the corresponding lithium ion conductivity is boosted by a factor of 2, which appears realistic in view of the simulations. This increase is most likely to occur within the outer layer (semi‐dilute brush region), as the molecular dynamics simulation suggest a slowdown of Li mobility closer to the particle surface.

### 2.6 Lithium Metal Anode Compatibility

**Figure 8:**
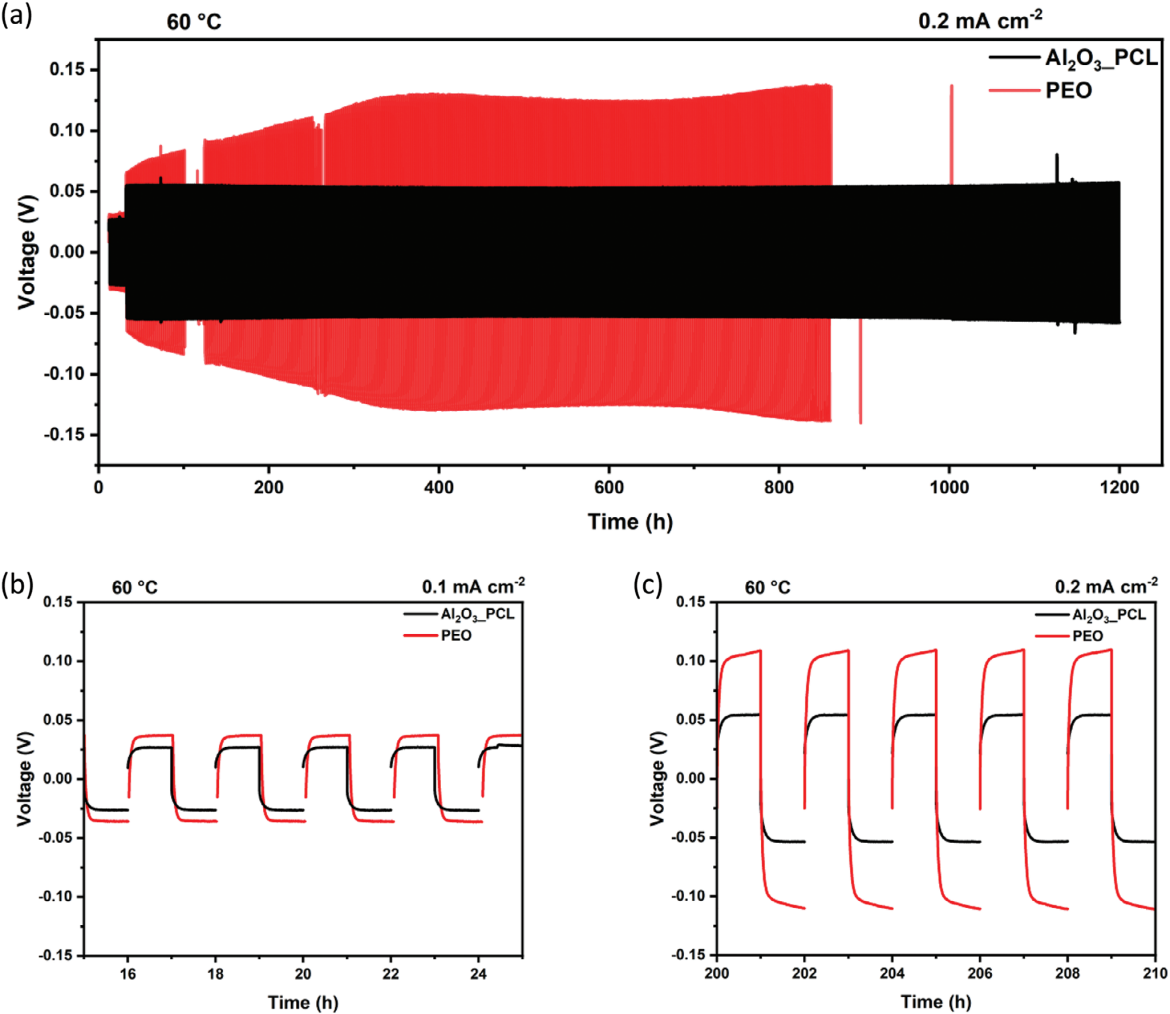
a) Long‐term lithium plating/stripping performance of Li||Li cells operated with Al_2_O_3__PCL and PEO reference electrolyte, and b) at a current density of 0.1 mA cm^−2^ and 60 °C, c) at a current density of 0.2 mA cm^−2^ and 60 °C. Here, the corresponding membrane thickness was 50–60 µm for both Al_2_O_3__PCL and PEO.

To classify Al_2_O_3__PCL as viable all‐dry solid electrolyte suitable for operation in lithium metal‐based batteries, electrochemical investigations of Al_2_O_3__PCL in Li||Li cells were performed, also accounting for reversible Li deposition and inventory as well as capability for fast charge application. Hence, galvanostatic Li metal stripping and plating experiments at current densities of 0.1 up to 1.1 mA cm^−2^ were done (Figure S7, Supporting Information), in each case keeping the time for Li metal stripping and plating constant at 1 h. As the actually applied current density increases, so does the overvoltage in the cells, and at a rather high current density of 1.1 mA cm^−2^, a dramatic increase in overvoltage is noticed. It is assumed that at this current density, depletion of lithium ions near the electrode surfaces and concentration polarization across the electrolyte occurred. Consequently, the limiting current density for operation of Al_2_O_3__PCL amounts to 1 mA cm^−2^, which is comparably high for fully dry polymer electrolytes.^[^ ^10^, ^48^, ^49^ ^]^ However, we note that for polymer electrolytes in general, even further improvement (>3 mA cm^−2^) is needed to increase the suitability for practical applications.^[^ ^50^ ^]^ Since a reversible lithium deposition and formation of robust SEI layers are of particular importance for potential longevity of polymer‐based cells, long‐term lithium stripping/plating tests were performed in Li||Li cells (**Figure** **8**).

Note that ten cycles at a current density of 0.1 mA cm^−2^ were performed, while for consideration of long‐term stability, the current density upon lithium stripping/plating was increased to 0.2 mA cm^−2^. In both cases, the time for stripping and plating was 1 h each, reflecting that subsequent NMC||Li cells were charged and discharged at C‐rates of up to 1 C. Figure 8b,c displays typical voltage curves as a function of time for Li||Li cells operated with Al_2_O_3__PCL and PEO reference electrolyte. After formation, the overvoltage of lithium deposition in case of Al_2_O_3__PCL hybrid electrolyte rises to 55 mV, which is twice as high compared to lithium deposition at 0.1 mA cm^−2^ (27 mV), in agreement with Ohm's law. Both systems were cycled for >1000 h (Figure 8a), equivalent to 500 cycles at a rate of 1 C, corresponding to a theoretical cathode capacity of 0.2 mAh cm^−2^. In comparison to other completely dry and solid polymer/oxide electrolyte systems in the literature (Table S4, Supporting Information) and in comparison to previous work, the number of cycles and the current density were considerably increased.^[^ ^6^, ^10^ ^]^ Al_2_O_3__PCL exhibits cycling at very stable overvoltage even after operation for 1200 h, whereas the cells with PEO reference demonstrate a higher and less robust overvoltage evolution (initially 64 mV at 0.2 mA cm^−2^) and cell failure evident at cycle 850. Indeed, the continuous increase of overvoltage in case of PEO represents insufficient SEI layer formation,^[^ ^51^ ^]^ while the shape of the voltage curve clearly demonstrates arcing behavior for cells operated with PEO, reflecting an occurrence of concentration polarization. In contrast, cells cycled with Al_2_O_3__PCL hybrid electrolyte exhibit a more rectangular shape of the voltage curve, suggesting that charge carrier diffusion is less limited at these current rates, in agreement with a significantly higher lithium transference number for Al_2_O_3__PCL electrolytes compared to the PEO reference.^[^ ^38^, ^39^ ^]^

### 2.7 Interfacial Charge Transfer

**Figure 9:**
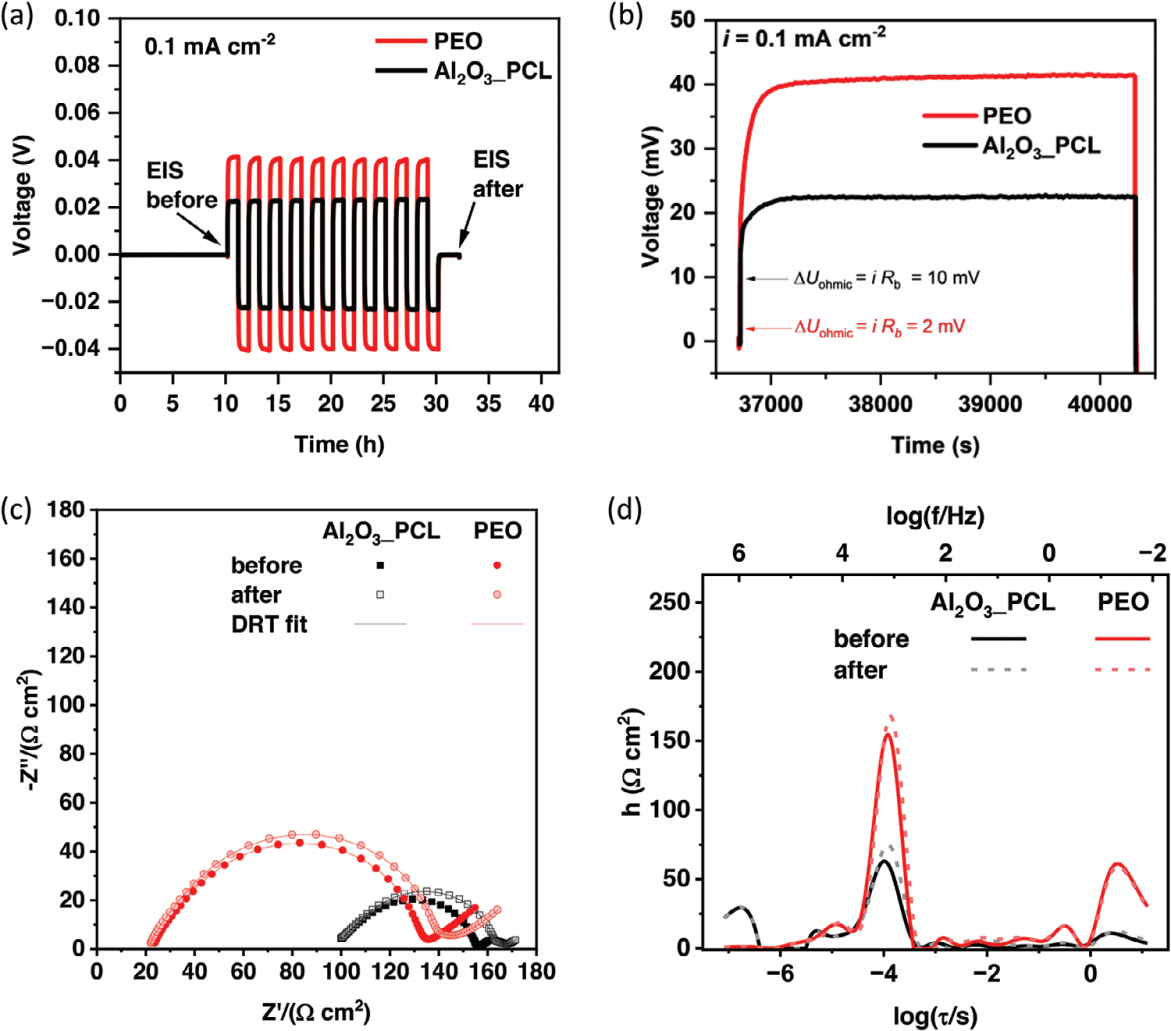
a) Constant current polarization of Li||Li cells containing PEO and Al_2_O_3__PCL hybrid electrolytes at a current density of 0.1 mA cm^−2^ and a temperature of 60 °C. b) Inset of the first polarization step; and c) impedance spectra of cells before and after plating/stripping experiments. d) Corresponding DRT spectra.

The interfacial charge transfer is evaluated by electrochemical impedance spectroscopy (EIS) and distribution of relaxation times (DRT) analysis. Impedance spectra of Li||Li cells were recorded before and after ten plating/stripping cycles at a current density of 0.1 mA cm^−2^ (0.1 mAh cm^−2^) at a temperature of 60 °C (**Figure** **9**c). The high frequency impedance (>100 kHz) corresponds to the bulk resistance of the respective electrolyte, that scales inversely with the ionic conductivity, and is substantially lower in case of PEO. At a membrane thickness of 54 µm for Al_2_O_3__PCL and 48 µm for PEO, the derived ionic conductivities are 5.4⋅10^−5^ and 2.1⋅10^−4^ S cm^−1^ for Al_2_O_3__PCL and PEO (**Table** **1**), respectively, and thus in good agreement with the values obtained with blocking electrodes. Remarkably, the interfacial resistances of Al_2_O_3__PCL containing cells is half that of the PEO containing cells, eventually contributing to the favorably low overvoltage of 22 mV compared to 41 mV (Figure 9a). The Ohmic drop (IR drop) is proportional to the bulk resistance and accounts for almost half of the overvoltage for Al_2_O_3__PCL (10 mV). In case of cells with PEO, most of the observed overvoltage originates from interfacial and mass transfer related resistances, as the Ohmic drop is only 2 mV. The higher mass transfer resistance for cells operated with PEO is also visible in the Nyquist and DRT plots, as the typical Warburg‐type impedance is much more pronounced compared to Al_2_O_3__PCL‐based cells.

**Table 1:** Bulk resistances, ionic conductivities, interfacial resistances and their corresponding characteristic time constants of Li||Li cells containing PEO and Al_2_O_3__PCL.

	Li|PEO|Li	Li|Al_2_O_3__PCL|Li
Bulk resistance R_b_ (Ω cm^2^)	23	100
Ionic conductivity σ (S cm^−1^)	2.1 × 10^−4^	5.4 × 10^−5^
Interfacial resistance R_int_ (Ω cm^2^)	110	55
Characteristic time constant τ (*s*)	1.0 × 10^−4^	1.0 × 10^−4^

The interfacial and bulk resistances, corresponding time constants and ionic conductivities are collected in Table 1. The values for the interfacial resistances were derived by integrating the area of the DRT peaks between 10^−3^ and 10^−6^ s. With 55 Ω cm^2^, the interfacial resistance of the Li|Al_2_O_3__PCL|Li cell is relatively low compared to other polymer electrolytes.^[^ ^29^ ^]^ For comparison, a benchmark value of 40 Ω cm^2^ was defined for inorganic solid state electrolytes to sustain charging rates of 1 C.^[^ ^52^ ^]^ Bulk resistances represent the sum of the series resistances obtained from DRT analysis and the integrated area of DRT spectra at time constants <10^−6^ s.

### 2.8 Cell Cycling Performance

**Figure 10:**
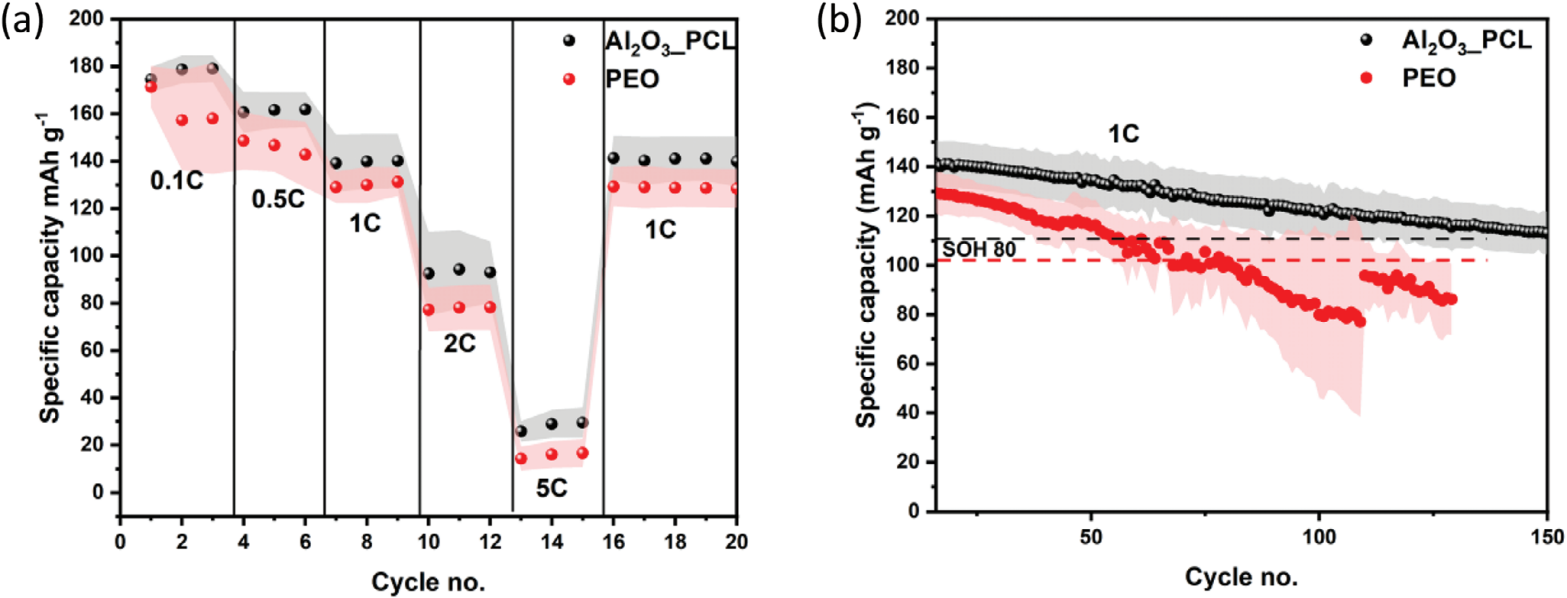
a) C‐rate performance at 0.1 C, 0.5 C, 1 C, 2 C, and 5 C; and b) long‐term cycling performance at 1 C of NMC_622_||Li cells with Al_2_O_3__PCL and PEO separator, operated at 60 °C; dotted lines mark SOH 80 condition. The cathode active mass loading was 1.7 mg cm^−2^, a lithium foil with thickness of 50 µm was utilized as anode, while the electrolyte membrane thickness ranged between 50 and 60 µm, respectively.

The respective C‐rate and electrochemical cycling performance of NMC_622_||Li cells operated with Al_2_O_3__PCL was determined at 60 °C (**Figure** **10**). For formation of the cells, a rate of 0.1 C for three cycles and a rate of 0.5 C for three cycles were applied, following a previously defined protocol. Subsequently, to establish rate performance and suitability for faster charge conditions, three cycles each at 1 C, 2 C, and 5 C were applied to the NMC_622_||Li cells, while the long‐term cycling was monitored at a rate of 1 C. In addition to Al_2_O_3__PCL and PEO, the electrochemical performance of cells cycled with Al_2_O_3__PCL_onlymix separator was obtained. In the latter case, as shown in (Figure S8, Supporting Information) all cells with Al_2_O_3__PCL_onlymix shorted after a few hours during the resting phase, attributed to the rather poor mechanical properties of linear PCL, where the simple addition of 20 wt.% Al_2_O_3_ nano‐particles as ceramic filler is insufficient to stabilize the resulting electrolyte membrane. The C‐rate test reveals that the achievable specific capacities of cells in case of Al_2_O_3__PCL are higher than those with PEO at all C‐rates. Even after straining of the cells at higher C‐rates of up to 5 C, it can be seen that the initial specific capacity of 140 mAh g^−1^ for cells operated with Al_2_O_3__PCL separator can be regained at rates of 1 C, clearly illustrating the electrochemical stability and application potential of the introduced Al_2_O_3__PCL hybrid electrolyte. In case of PEO, it is noticeable that the standard deviation of the achievable specific capacity during cell formation was significantly higher than for the remaining cycles, reflecting poor stability of PEO against NMC cathodes at higher cell voltage (of up to 4.3 V).^[^ ^53^, ^54^, ^55^ ^]^ Indeed, long‐term cycling of NMC_622_||Li cells at 1 C demonstrates that Al_2_O_3__PCL is much more electrochemically stable than PEO, and the initially achieved specific capacity is 12% higher than that of the cells with PEO electrolyte. The capacity drop for the PEO cells at 0.1 C could be attributed to parasitic side reactions with the NMC cathode material, which is likely stabilized in the short term by the formation of a CEI, as observed in the subsequent cycles. Even after more than 100 cycles at 1 C, the capacity of cells cycled with Al_2_O_3__PCL is still above the state‐of‐health (SOH) 80, whereas the cells with PEO electrolyte suffer from strong capacity fading after a short operational time, reaching the limit of SOH 80 at the 60th cycle. The Coulombic efficiency at the 100th cycle for cells operated with Al_2_O_3__PCL amounts to >99% compared to merely 73.5% in case of PEO, in agreement with an occurrence of parasitic currents, likely caused by PEO decomposition at the electrodes.^[^ ^53^, ^56^ ^]^

**Figure 11:**
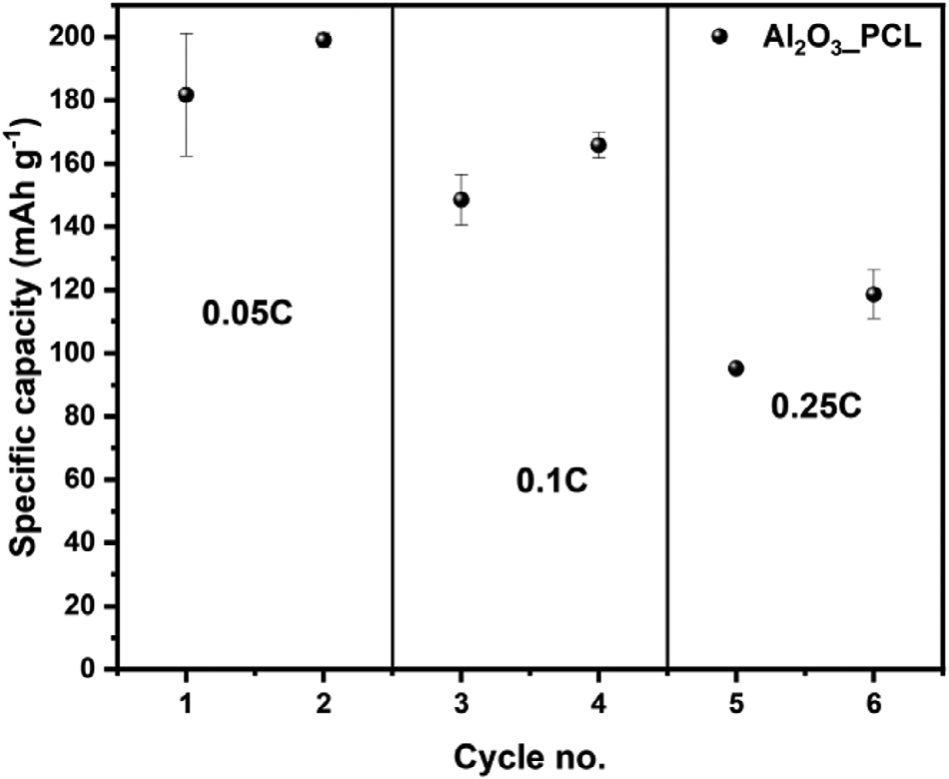
C‐rate performance at rates of 0.05 C, 0.1 C, 0.25 C of NMC_622_||Li cells operated with Al_2_O_3__PCL separator, at a temperature of 60 °C. The cathode active mass loading was adjusted to 6 mg cm^−2^, a lithium foil with thickness of 50 µm was utilized as anode, while the electrolyte membrane thickness ranged between 50 and 60 µm, respectively.

In addition to the proof‐of‐concept lower mass loadings (≤ 2 mg cm^−2^), the suitability of the all‐dry solid hybrid polymer electrolytes to potentially operate with higher mass loading cathodes was explored based on NMC_622_||Li cells and cathodes with mass loadings of 6 mg cm^−2^. In order to contact the higher mass‐loading cathodes and cycle cells with Al_2_O_3__PCL hybrid electrolytes, a mixture of linear PCL and conductive lithium salt (LiTFSI) was melted and infiltrated into the corresponding cathodes. The results of the C‐rate test are shown in **Figure** **11**.

Upon infiltration with linear PCL, it was possible to sufficiently contact the cathodes, that at a C‐rates of up to 0.1 C, specific capacities of 150 mAh g^−1^ could be achieved, reflecting almost full discharge of the cathodes, while at higher C‐rates of 0.25 C, merely 100 mAh g^−1^ discharge capacity was obtained. The drop in specific capacity in case of the all‐dry polymer cell system likely results from charge carrier kinetic limitation within the porous composite cathodes and across electrolyte‐|electrode interfaces, as well as presence of internal voids that are otherwise filled by flowable liquid electrolytes.^[^ ^57^, ^58^ ^]^ The infiltration process could be further optimized to achieve more complete contacts with the active material particles. By using polymers with a lower viscosity in the melt, the polymer could flow better into the cavities of the cathode during melting. Note that the commercial cathodes were already calendered prior to cell assembly and thus were compressed; upon infiltrating the polymer upstream and then calendering, the voids in the cathode would initially be larger, which should facilitate pronounced polymer penetration. In case of all‐dry solid electrolyte systems, application of cathodes with higher mass loadings is anything but trivial, as also documented based on data available from current literature and patents (Table S2, Supporting Information), where higher cathode mass loadings were realized only when invoking non‐volatile flowable components such as PCL‐based oligomers or other plasticizers.^[^ ^10^ ^]^ An alternative to non‐volatile flowable catholytes could be tailored composite cathodes whose intrinsic charge carrier transport ability is notably boosted by the presence of argyrodites (that is, a solid electrolyte is mixed with cathode active material during cathode manufacture and processing). The latter may sufficiently counteract kinetic limitations of charge carrier transport, considering the exceptionally good ionic conductivity (of up to 25⋅10^−3^ S cm^−1^).^[^ ^59^, ^60^ ^]^ Indeed, preliminary electrochemical cycling of NMC‐Ni82||Li cells operated with argyrodite‐based composite cathodes with mass loadings of 6–7 mg cm^−2^ and Al_2_O_3__PCL electrolytes were already carried out, yielding specific capacities of 70 mAh g^−1^ at C‐rates of 0.1 C, temperature of 60 °C and a pressure of 12.5 MPa. Despite invoking so‐called “press cells”, the up to now unfavorably low achieved discharge capacity is yet attributed to cell operation at lower pressure conditions, which otherwise typically amount to up to 50 MPa in case of argyrodites.^[^ ^61^, ^62^, ^63^ ^]^ Such high‐pressure conditions, however, appear too demanding even for the introduced mechanically reinforced Al_2_O_3__PCL hybrid polymer electrolytes, in principle reflecting mechanical weakness of polymer‐based compared to available inorganic electrolytes. Nevertheless, no degradation of Al_2_O_3__PCl electrolytes in the presence of argyrodites was noticed, emphasizing versatility of the hybrid electrolyte. While the required cell pressure could possibly be reduced by further optimizing the respective cathode composition (which will be investigated in subsequent work), the demonstrated compatibility of Al_2_O_3__PCL with Li metal anodes in principle could facilitate future hybrid NMC||Li cell designs that are operated with either argyrodite or halide‐based electrolytes and an Al_2_O_3__PCL protective coating on the lithium metal electrode.

## 3 Conclusion

In this work, hybrid electrolytes Al_2_O_3__PCL, based on poly(caprolactone) polymer and aluminum oxide nano‐particles, are introduced and prepared by a one‐pot grafting reaction of Al_2_O_3_ nano‐particles with caprolactone precursor. The solvent‐free polymerization of Al_2_O_3__PCL is straightforward and suitable for up‐scaling to a kg batch size. Poly(caprolactone) in principle is biodegradable, environmentally friendly and affordable, as are commercially available Al_2_O_3_ nano‐particles, rendering the hybrid electrolyte potentially sustainable. Al_2_O_3__PCL could be produced as all‐dry, flexible and mechanically robust, free‐standing membrane with a thickness of 50 µm, eventually allowing polymer processing by extrusion and heated calendering for automated production of even thinner polymer membranes. Indeed, PCL‐based electrolytes are an attractive alternative to PEO‐based electrolytes, attributed to higher lithium transference numbers and lithium ion conductivities compared to PEO‐based materials. Lithium metal could be reversibly stripped and plated in Li||Li cells at comparatively high current densities of up to 0.2 mA cm^−2^ for more than 1200 h, where Al_2_O_3__PCL exhibited a favorably low overvoltage of 54 mA. The mechanical weakness of pristine PCL‐based systems could be eliminated by grafting, that is formation of covalent bonds among Al_2_O_3_ nano‐particles and PCL. Grafting also showed in this case a decoupled behavior for the ionic conductivity from mechanical stability of the hybrid polymer electrolyte, enabling better mechanical stability and increased ionic conductivity. The latter results from a local increase in ionic conductivity in areas of the grafted chains in the vicinity of the ceramic particle surfaces. The determined electrochemical stability window (ESW > 5 V) enabled cycling of Al_2_O_3__PCL against higher voltage cathodes materials such as NMC_622_, yielding SOH >80% for more than 140 cycles in NMC_622_||Li cells at C‐rates of up to 1 C. In summary, this work provides a proof‐of‐concept for exploitation of PCL‐grafted ceramic particles as hybrid electrolyte in lithium metal batteries. Though the Al_2_O_3__PCL is still not a fully optimized system in terms of particle/polymer ratio, grafting density, and other conditions, the significant potential of these new type of hybrid electrolytes is evident. The described concept can be expanded by invoking differently conditioned ceramic materials with variable particle sizes and shapes, allowing for a variety of range PCL‐based hybrid materials. Note that key factors such as particle size, chain length of the grafted polymer chains, chain length of the polymers, particle/polymer ratio, grafting density on the particle surface, and many others affect the achievable mechanical and electrochemical properties (including ionic conductivity) of the grafted hybrid electrolytes, and further computational efforts could corroborate identification of promising hybrid electrolytes with tailored properties. Since the presented data of this work emphasizes the potential of polymer‐grafted ceramic particle hybrid electrolytes, it should be considered as an incentive to further explore the concept of grafting for the design of hybrid electrolytes based on synergistic effects that are readily up‐scalable and suitable for operation in metal‐based cell chemistries. We emphasize, that even under more challenging conditions, this approach will pave ways for new beyond‐PEO polymer based solid state electrolytes.

## Conflict of Interest

The authors declare no conflict of interest.

## Author Contributions

The manuscript was written through contributions of all authors. All authors have given approval to the final version of the manuscript. F.S. performed Conceptualization, Investigation, Wrote the Original Draft. A.K., P.L., A.B., F.K., P.G., M.M.M. performed Investigation. M.C. and G.S. performed Simulations. D.D. and T.D. performed Simulations and Editing. A.H., A.L., M.W., and G.B. performed Supervision, Funding Acquisition, Wrote the Original Draft, Review & Edited the final manuscript.

## Supporting information

Supporting Information

## Data Availability

The data that support the findings of this study are available from the corresponding author upon reasonable request.
