# Supplementary material for: Synergistic Enhancement of Mechanical and Electrochemical Properties in Grafted Polymer/Oxide Hybrid Electrolytes
Source: Small. 2024 Aug 26;20(47):2404537. doi: 10.1002/smll.202404537 (PMC11579982; doi:10.1002/smll.202404537)
Supplement: Supplementary file 1 — Supporting Information [file SMLL-20-2404537-s001.docx]

Supporting Information

Synergistic Enhancement of Mechanical and Electrochemical Properties in a Grafted Polymer/Oxide Hybrid Electrolyte

Felix Scharf^1^, Annalena Krude^1^, Peter Lennartz^1^, Moritz Clausnitzer^3^, Gourav Shukla^4^, Annika Buchheit^1^, Fabian Kempe^1^, Diddo Diddens^1,4^, Pascal Glomb^1^, Melanie M. Mitchell^1^, Timo Danner^3^, Andreas Heuer^1,4^, Arnulf Latz^3^, Martin Winter^1,2^, Gunther Brunklaus*^1^

^1^ Forschungszentrum Jülich GmbH, Helmholtz-Institute Münster, IMD-4,

Corrensstr. 48, 48149 Münster, Germany

^2^MEET Battery Research Center, Institut für Physikalische Chemie

University of Münster

Corrensstraße 46, 48149 Münster, Germany

^3^ Deutsches Zentrum für Luft- und Raumfahrt (DLR)

Helmholtz Institut Ulm (HIU) – Institut für Technische Thermodynamik Computergestützte Elektrochemie

Helmholtzstraße 11, 89081 Ulm Germany

^4^ Universität Münster Institut für Physikalische Chemie,

Correnstr. 28/30, 48149 Münster, Germany

**Table of Contents**

[**1.** **Experimental** 2](#_Toc166255340)

[**2.** **Figures on electrochemical and physical investigations** 12](#_Toc166255341)

[**3.** **Upscaling of Al_2_O_3__PCL with a 2L Reactor** 15](#_Toc166255342)

[**4.** **Patent research for start-ups working with polymer electrolyte** 16](#_Toc166255343)

[**5.** **Cost comparison of Al_2_O_3__PCL and PEO electrolytes** 17](#_Toc166255344)

[**6.** **Table of Electrochemical performance of a variety of mixed and grafted polymer/oxide hybrid electrolytes** 18](#_Toc166255345)

[**7.** **References** 20](#_Toc166255346)

# **Experimental**

Experimental

**Material**

ε-Caprolactone (97%, Sigma Aldrich), Aluminium Oxide Nanoparticles (MSE Supplies), tin(II) 2-ethylhexanoate (92.5-100.0%, Sigma Aldrich), tetrahydrofuran (THF) (≥ 99.0%, Sigma Aldrich), toluene (99.8%, anhydrous, Sigma Aldrich), methanol (≥ 99.8%, Sigma Aldrich), benzophenone (Sigma Aldrich), LiNi0.6Co0.2Mn0.2O2 (NMC622) (BASF TODA Battery Materials LLC), lithium bis(trifluoromethanesulfonyl)imide (LiTFSI) (Sigma Aldrich), Super C65 (Imerys Graphite & Carbon), polyvinylidene difluoride (PVdF) (Solef® 5130, Solvay), N-methyl-2-pyrrolidone (NMP) (99.5%, Sigma Aldrich), lithium metal (50 μm, Honjo Chemical Corporation), Poly(ethylene oxide) (PEO) (MW=5,000,000 g mol-1 , Sigma Aldrich)

**Synthesis of Al2O3-PCL**

Under inert gas, 3 g of aluminum oxide powder (300nm) and 12ml of ε-caprolactone were placed in a pre-dried 100 ml round bottom flask. The mixture was stirred with a magnetic stirrer on a hot plate and heated to 110 °C. Then 0.5 ml tin(II) 2-ethylhexanoate was added to the mixture under nitrogen using a syringe to start the polymerization. The mixture was stirred at 110 °C for a further 24 hours. After the reaction time of 24 h, the mixture was cooled to 25 °C and 90 ml of toluene was added to the round bottom flask. The dissolved Al_2_O_3__PCL was then transferred stepwise into a 650 ml beaker filled with methanol using a pipette. This caused the Al_2_O_3__PCL to precipitate. After the polymer solution had been completely transferred to the beaker, the methanol was decanted from the Al_2_O_3__PCL. Another 300 ml of methanol was added to the Al_2_O_3__PCL and stirred for about 5 minutes; this was repeated one more time. The Al_2_O_3__PCL was then pre-dried in a crystallization dish at 40 °C under vacuum. After 24 hours, the Al_2_O_3__PCL was dried with the aid of a turbopump at 40 °C up to a pressure of 10^-7^ mbar. The yield of the reaction is 95%.

**Upscaling**

To demonstrate the scalability of the synthesis, a 500 g batch of Al_2_O_3__PCL with approx. 10wt% Al_2_O_3_ particles was prepared. For this purpose, 450 g ε-caprolactone was placed in a 2L reactor (IKA EasySyn 2000). Under stirring and nitrogen, 55 g Al_2_O_3_ powder was added to the monomer. The reactor was heated to 110 °C. After reaching the temperature, 9.1 ml tin(II) 2-ethylhexanoate was added to the mixture using a syringe. The reaction lasted 24 hours and was carried out in a nitrogen atmosphere. After the reaction time, the reactor was cooled to 25 °C and 1.5 L of toluene was added to the reactor. The dissolved Al_2_O_3__PCL was gradually removed from the reactor and precipitated in methanol. The Al_2_O_3__PCL was washed once more with methanol and then dried in a vacuum oven at 40 °C for 48 h.

**Membrane preparation**

The processing and production of the membrane was carried out in a dry room.

**Al_2_O_3__PCL:** The pre-dried materials such as Al_2_O_3__PCL, LITFSI and benzophenone were weighed into a Teflon dish. The Teflon dish was placed on a hot plate at 140 °C, the Al_2_O_3__PCL and the benzophenone started to melt and the three materials were mixed with a spatula. After all materials had melted, the mixture was formed to a ball and was placed between two Mylar films (100 μm, silicone coated, PPI Adhesive Products GmbH) and pressed with the hot press (Polystst 200T) at 140 °C and a pressure of 10 bar to 25 bar. After two minutes, the membrane was removed from the hot press and formed back into a ball. This process was repeated five times. The polymer ball was then sealed in a pouchbag under vacuum. The pouchbag with the polymer was then placed in an oven at 100 °C for 12 hours. The polymer ball was then removed from the pouchbag and positioned between two Mylar films. The polymer ball with the Mylar films was then placed in the hot press and pressed at 140 °C for 10 min at 5 bar, 5 min at 10 bar, 2 min at 50 bar, 2 min at 100 bar and 5 min at 150 bar. The surface of the membrane was then cross-linked using UV radiation (UVACUBE 100, Dr. Hönle AG) for 5 min per side.

**Al_2_O_3__PCL_onlymix:** The pre-dried materials such as LPCL, LITFSI and benzophenone were weighed in a Teflon dish. The Teflon dish was placed on a hot plate at 100 °C, the LPCL and the benzophenone started to melt and the three materials were mixed with a spatula. After all materials had melted, the mixture the mixture was formed to a ball and was placed between two Mylar films (100 μm, silicone coated, PPI Adhesive Products GmbH) and pressed with the hot press (Polystst 200T) at 100 °C and a pressure of 10 bar to 25 bar. After two minutes, the membrane was removed from the hot press and formed back into a ball. This process was repeated five times. The polymer ball was then sealed in a pouchbag under vacuum. The pouchbag with the polymer was then placed in an oven at 100 °C for 12 hours. The polymer ball was then removed from the pouchbag and positioned between two Mylar films. The polymer ball with the Mylar films was then placed in the hot press and pressed at 100°C for 10 min. at 5 bar, 5 min. at 10 bar and 5 min. at 50 bar. The pressure and temperature could be set lower than for Al_2_O_3__PCL, as the LPCL is significantly softer and flows better in the melt. The membrane was then cross-linked using UV radiation (UVACUBE 100, Dr. Hönle AG) for 5 min. per side.

**PEO:** The pre-dried materials such as PEO, LITFSI and benzophenone were weighed in a Teflon dish. The Teflon dish was placed on a hot plate at 100 °C, the LPCL and the benzophenone started to melt and the three materials were mixed with a spatula. After all materials had melted, the mixture the mixture was formed to a ball and was placed between two Mylar films (100 μm, silicone coated, PPI Adhesive Products GmbH) and pressed with the hot press (Polystst 200T) at 100 °C and a pressure of 10 bar to 25 bar. After two minutes, the membrane was removed from the hot press and formed back into a ball. This process was repeated five times. The polymer ball was then sealed in a pouchbag under vacuum. The pouchbag with the polymer was then placed in an oven at 100 °C for 12 hours. The polymer ball was then removed from the pouchbag and positioned between two Mylar films. The polymer ball with the Mylar films was then placed in the hot press and pressed at 100 °C for 10 min. at 5 bar, 5 min. at 10 bar, 2 min. at 50 bar and 5 min. at 100 bar. The membrane was then cross-linked using UV radiation (UVACUBE 100, Dr. Hönle AG) for 5 min per side.

**Cathodes preparation**

NMC_622_ electrodes consisting of 90 wt.% (1.854 g) NMC_622_-Powder (BASF TODA Battery Materials LLC), 7 wt.% (0.144 g) Carbon Black (Super C65, Imerys Graphite & Carbon) and 3 wt.% (0.062 g) PVdF (Solef® 5130, Solvay) were produced by first dissolving PVdF in 4 ml (3,883 g) NMP (99.5%, Sigma Aldrich) using an ARM-310 ThinkyMixer (THINKY) for 20 Minutes at 2000 RPM. Carbon Black and NMC622 were subsequently added to the mixture and homogenized with the ThinkyMixer for 20 min. at 2000 RPM and 10 min. at 300 RPM to get rid of any remaining air bubbles within the mixture. The slurry was cast onto previously cleaned aluminum foil (20 µm, Evonik Industries) by using a doctor blade (ZUA 2000 Universal Applicator, ZEHNTER GmbH) with a wet coating thickness of 40 – 50 µm and an Automatic Film Applicator (1133 N, Sheen Instruments) with a speed of 50 mm s^−1^. The electrode sheets were dried in an oven (Binder GmbH) at 80 °C for 2 hours before drying under reduced pressure (10^-3^ mbar) over night at 110 °C. This step was followed by calendaring to a thickness of ~ 34 – 38 µm, and punching into circular electrodes using a Hohsen electrode puncher (Hohsen Corp.) with a diameter of Ø = 12 mm and further dried again over night at reduced pressure (10^-5^ mbar) before being used for cell production. The produced cathodes are corresponding to ~ 1.7 mg cm^-2^ CAM.

**Cell fabrication**

**Full cells:** Model CR2032 coin cells were used. The cathode had a diameter of 12 mm, the electrolyte of 14 mm and the lithium metal of 14 mm.

**Symmetrical cells Li||Li:** Model CR2032 coin cells were used. The lithium had a diameter of 10 mm and the electrolyte of 12 mm.

**Blocking electrodes:** Model CR2032 coin cells were used. A spacer ring made of Mylar foil with an outer diameter of 16 mm and an inner diameter of 10 mm was positioned between the stainless steel electrodes. The electrolyte with a diameter of 10 mm was positioned in this spacer ring. Spacer ring and electrolyte had the same thickness.

2.1. Electrochemical characterization

All the cells were prepared and assembled in a dry room. Model CR2032 coin cells were used.

**EIS conductivity**: Symmetrical stainless steel cells with the polymeric electrolyte in between were used for conductivity determination (CR2032 coin cells). All measurements were performed in a temperature range between 20 and 70 °C in a climatic chamber (Binder MK 053), temperature fluctuations did not exceed ±0.02 K. For determination of the cell impedance, logarithmic frequency scans of 50 steps from 1−10^6^ Hz with an alternating voltage of 10 mV were performed. The experiments were performed on an Multi Autolab M204 equipped with FRA32M-module (Deutsche Metrohm) and the data were collected with NOVA software 2.1.4 (Metrohm Autolab B.V.). The conductivity was calculated according equation S1.

Ep. S1: $\sigma=\frac{1}{R}\left( \frac{d}{\pi r^{2}} \right)$

**EIS of Li||Li cells and Distribution of Relaxation Times**

Electrochemical impedance spectra of Li||Li cells were acquired before and after polarization with a current density of 0.1 mA cm^-2^. The AC amplitude was 10 mV at OCV (i.e. 0 V vs. Li/Li^+^) and the applied frequency window was 1 MHz to 100 mHz. The obtained resistances were normalized to the electrode area (multiplication with 0.785 cm^2^). The impedance spectra were analyzed with a Distribution of Relaxation Times (DRT) using a custom-made Python script. For optimal resolution, the number of time constants was chosen as six-fold the number of experimental data points. A regularization parameter of $\lambda=0.2$ was chosen as a good compromise between smoothening noise and increasing the residual norm of the fit. A series resistor and inductor were included in the fitting model to further enhance the goodness of the fit.

**Transference number**

Transference numbers were measured according to Bruce-Vincent method.^[1,2]^ Therefore, symmetrical Li||Li cells with the polymeric electrolyte were built (CR2032 coin cells). All measurements were performed in a temperature range between 40 and 80 °C in a climatic chamber (Binder MK 053), temperature fluctuations did not exceed ±0.02 K. The cells were equilibrated for at least 20 h, meanwhile the impedance of the polymer/lithium boundary was monitored in order to ensure the formation of a stable SEI. For determination of transference numbers, a constant DC voltage of 10 mV (ΔV) was applied to the cell and the time dependent current was monitored, the initial current is denoted as *I*_0_ and the final steady state current is denoted as *I*_ss_ in the following. The cell impedance was analysed before and after each single DC polarization step by AC impedance measurements. Therefore, logarithmic frequency scans of 50 steps from 1−10^6^ Hz with an alternating voltage of 10 mV were performed. The ohmic part of the electrolyte impedance and the lithium/electrolyte interface impedance is denoted as R_el,0_ and *R_Li,_*_ss_ for the sample before polarization and after polarization in steady state, respectively. Complete transference experiments including the polarization and intermediate impedance measurements were performed on an Multi Autolab M204 equipped with FRA32M-module (Deutsche Metrohm) and the data were collected with NOVA software 2.1.4 (Metrohm Autolab B.V.). Calculation of transference numbers were done according to equation 1.^[1,2]^

Eq. S2: $t_{+}=\frac{R_{el,0} I_{ss}}{\Delta V-R_{Li,ss} I_{ss}}$

**Linear sweep voltammetry**

LSV experiments were carried out with an Autolab PGSTAT302 N (Metrohm) potentiostat at 60 °C, data were collected with the software NOVA 2.1.4 (Metrohm Autolab B.V.). Samples were measured in a three electrode setup in Swagelok cells, whereas Lithium served as the counter and reference electrode. For reductive scan, Nickel was used as working electrode, for oxidative scan Platinum served as the working electrode respectively. Reductive scan was performed between OCV and -0.4 V vs. Li│Li^+^ or until lithium deposition, oxidative scan was performed between OCV and 7 V vs. Li│Li^+^ or the current exceeds 10^-5^ A.

**Stripping/Plating**

**Lithium plating/stripping experiments** were conducted on a Maccor (Series 4000 Battery Tester). Symmetric Li||Li cells were applied a current density of 0.1 mA cm^-2^ for formation and 0.2 mA cm^-2^ for long-term, plating/stripping for 1 hour per half cycle.

**Cycling**
NMC622||Li cells with a CAM of 1.7 mg cm^-2^ were cycled in a Memmert oven at 60 °C with a Maccor (Series 4000 Battery Tester). NMC622|SHE|Li full cells were assembled using 14 mm (in diameter) of the SHE membrane, 14 mm of Li metal and 12 mm of cathodes. The full cells were aged at 60 °C for 12 h before cycling. The formation of the cells was carried out with three 0.1 C and three 0.5 C steps, followed by a C rate test with three 1 C, three 2 C and three 5 C steps. Long-term cycling was then performed with 1 C. All cells were cycled in the voltage range from 3 to 4.3 V (constant current, CC) with constant voltage charging (CV) at 4.3 V either for 30 min or until charge current smaller than 0.05 C.

NMC622||Li cells with a CAM of 6 mg cm^-2^ were cycled in a Memmert oven at 60°C with a Maccor (Series 4000 Battery Tester). NMC622|SHE|Li full cells were assembled using 12 mm (in diameter) of the SHE membrane, 10 mm of Li metal and 10 mm of cathodes. The full cells were aged at 60 °C for 12 h before cycling. The formation of the cells was carried out with two 0.05 C steps, followed by a C rate test with three 0.05, three 0.1 C, three 0.25 C steps. All cells were cycled in the voltage range from 3 to 4.3 V (constant current, CC) with constant voltage charging (CV) at 4.3 V either for 30 min or until charge current smaller than 0.05 C.

NMC_83_/argyrodite||Li cells with a CAM of 6 – 7 mg cm^-2^ were cycled in a Binder (KT115) oven at 60 °C with a Biologic (VSP-300). NMC_83_|SHE|Li full cells were assembled using 10 mm (in diameter) of the SHE membrane, 9 mm of Li metal and 9 mm of cathodes. For electrochemical measurements a PEEK lined, airtight press cell with stainless steel stamps with a surface area of 0.785 cm^2^ as current collectors were used.^[3]^ Mit hilfe einer uniaxial press wurde ein Druck von 12.5 MPa auf den Zellstack ausgeübt. Die Zelle wurde dann bei diesem Druck mit einer C rate von 0.1 C bei 60 °C Zyklisiert. All cells were cycled in the voltage range from 3 to 4.3 V (constant current, CC) with constant voltage charging (CV) at 4.3 V either for 30 min or until charge current smaller than 0.05 C.

2.2. Rheological and thermal characterization

**Rheological characterization** was carried out on a Physica MCR 301 with CTD 450 and a measuring plate PP15 (Anton Paar). The shear rheological measurements were determined with an amplitude of γ = 0.1 % and in a frequency range of ω = 100 - 0.1 rad s^-1^. The contact pressure during the measurement was 1 N.

**Thermogravimetric analyses** (TGA) were conducted on a Discovery TGA 5500 device (TA Instruments). The samples were firstly heated up to 150 °C with a temperature ramp rate of 20 °C min^-1^, and then kept isothermally at 150 °C for 1 h (Helium flow rate: 25 mL min^-1^).

In the second experiment, the sample was heated to 900 °C with a temperature ramp rate of 10 °C min^-1^ (helium flow rate: 25 mL min^-1^).

**Differential scanning calorimetry** (DSC) measurements were performed on a heat flux calorimeter DSC 2500 from TA Instruments with liquid nitrogen cooling system (LN2P) and Tzero®‑technology. ~10 mg of sample was filled in an aluminum pan and hermetically sealed. With a heating rate of 10 K·min^‑1^ two cycles from ‑140 °C to 150 °C under N_2_ protection gas (50 mL·min^‑1^) were measured. The peak maxima were used to determine the melting point and the inflection point of the curves was used to determine the glass transition temperature.

**Size exclusion chromatography** (SEC) measurements were conducted on a 1260 Infinity GPC/SEC System (Agilent). Separation was carried out in one pre-column SDV 10 µm (8 mm × 50 mm) and three SDV 10 μm columns (8 mm × 300 mm) at 35 °C, using tetrahydrofuran (THF) as eluent (flow rate: 1 mL min-1). Detection was performed with a refractive index detector. A calibration kit from Agilent was used to calibrate the SEC (ReadyCal-Kit Poly(styrene) high.

**Continuum simulations:** Effective ionic conductivities are calculated using 3D microstructure-resolved continuum simulations. For these simulations, we rely on the simulation framework BEST (Battery and Electrochemistry Simulation Tool).^[4,5]^ Input parameters of the simulation are the conductivities of the electrolyte materials and the hybrid-SE microstructure. Starting from the experimentally determined bulk conductivities, a broad parameter space is considered to identify possible physical processes that contribute to the observed increase in conductivity of the grafted polymer system.

Figure S1 exemplarily shows an input geometry for our simulation study. Initially, spherical $\mathrm{Al}_{2}O_{3}$ particles were randomly distributed in a homogeneous $\mathrm{LPCL}$ matrix to replicate the resulting microstructure of the grafting procedure.

Based on the results of the MD simulations, we assume that the grafted polymer chains exhibit increased conductivity compared to the bulk polymer. The rise in local conductivity can be particularly attributed to the stretching of the polymer chains due to grafting and a correspondingly improved Li-ion transport along the chains. Additionally, interfacial effects can lead to enhanced conductivity in the interfacial regions of the ceramic particles.^[6,7]^ In our continuum simulations, we model the grafted polymer chains as a homogeneous layer around the $\mathrm{Al}_{2}O_{3}$ particles, defined by a layer thickness and an isotropic conductivity. Since $\mathrm{Al}_{2}O_{3}$ particles are passive fillers, they do not directly contribute to Li-ion transport in the hybrid electrolyte. The parameters used for the structure generation and continuum simulations are summarized in Table S1.

We calculate the effective ionic conductivity $\sigma_{\mathrm{eff}}$ of the electrolyte structures by solving the Poisson equation on the hybrid electrolyte microstructure. According to Eq. 1, the conductivity is calculated from the current density *i* at an applied voltage of *U*=1 V at the boundaries of the structures. *l* is the length of the electrolyte microstructure.

Eq. S3: $\boldsymbol{\sigma}_{\mathbf{eff}}\boldsymbol{=}\boldsymbol{l}\boldsymbol{\cdot}\frac{\boldsymbol{i}}{\boldsymbol{U}}$

**Table S1:** Parameters used for the effective conductivity simulations.

| **Symbol** | **Value** | **Unit** | **Short description** |
| --- | --- | --- | --- |
| Microstructure | | | |
| $d_{\mathrm{Al}_{2}O_{3}}$ | 300 | nm | $\mathrm{Al}_{2}O_{3}$ particle diameter |
| $\varphi_{\mathrm{Al}_{2}O_{3}}$ | 8% | - | Volume fraction of $\mathrm{Al}_{2}O_{3}$ |
| $L\times W\times H$ | $500\times500\times500$ | voxels | Dimensions of microstructure |
| $\Delta v$ | 50 | nm | Voxel size |
| Material parameters | | | |
| $\sigma_{\mathrm{LPCL}}$ | $3.3\cdot{10}^{-2}$ | mS/cm | Effective conductivity of LPCL at 60 °C |
| $\sigma_{LPCL,g}$ | Varies | mS/cm | Effective conductivity of grafted LPCL at 60 °C |
| $t_{LPCL,g}$ | Varies | nm | Thickness of grafted polymer layer |

| **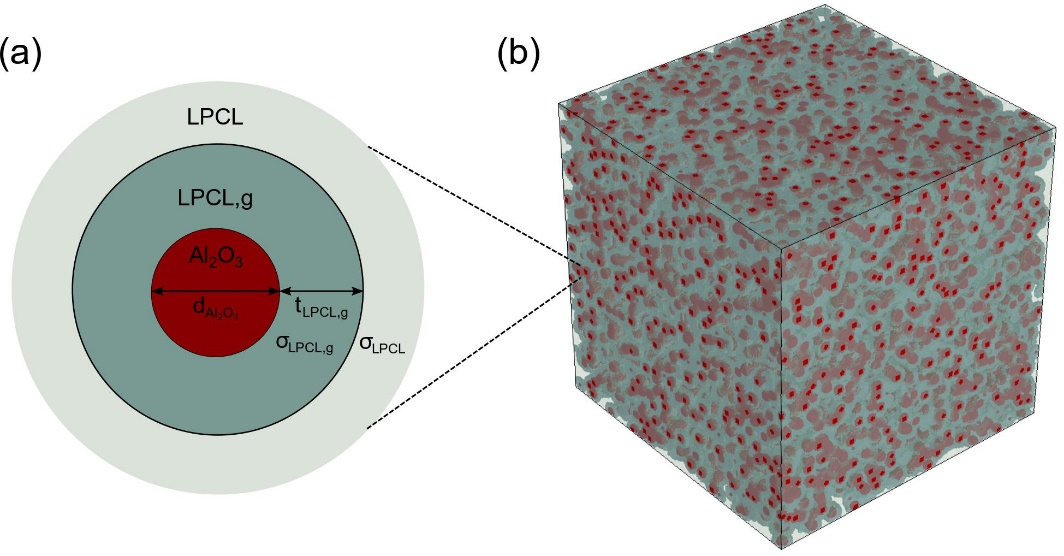** |
| --- |
| **Figure S1:** Generated microstructure for the effective conductivity simulations. (a) Overview of the relevant materials and parameters. The grafted polymer layer is defined by the conductivity $\sigma_{LPCL,g}$and thickness $t_{LPCL,g}$. (b) Exemplary 3D microstructure with $t_{LPCL,g}=200 nm$. For improved clarity, the size of the simulation geometry was reduced from $500\times500\times500$ to $50\times250\times250$ voxels. |

**Molecular Dynamics simulations:** The hybrid electrolyte consisted of polycaprolactone (PCL) polymers with a LiTFSI salt (ratio of 5:1) and an Al_2_O_3_ solid crystal with dimensions of 4.83281 x 8.30333 x 8.92388 Å (unit cell, crystal structure data retrieved from Materials Project for Al_2_O_3_ (mp-2254) from database version v2023.11.1,^[8]^ which was turned into a slab by replicating it (6 x 3 x 3) times in *x*, *y* and *z* direction, and by removing periodicity in *z* direction. Single chains with five monomers were stretched along the *z* axis, perpendicular to the Al_2_O_3_ surface, such that the oxygen-terminated end of the chain was close to the surface. These chains were then used to generate the grafted surfaces for different grafting densities. In our case, we have used two types of grafting densities, that is, 0.56 and 2.23 PCL chains per nm². To mimic the grafting, the oxygen atom of the OH-terminated end near the surface was fixed at a distance of 1 Å. Furthermore, the forcefield parameters (LJ parameters and charges) for the atoms of the OH group were turned to zero, such that these fixed points act as a dummy hinged point, that is, holding fixed the grafted polymer chain on one of its ends. The grafting procedure was applied to both surfaces of the Al_2_O_3_ slab. For the lower grafting density, 2x2 chains and for the medium grafting density 4x4 chains were used on either side of the Al_2_O_3_ slab. Additionally, the box was filled with free PCL chains on top of the grafted domains using fftool^[9]^ and Packmol^[10]^ such that the systems in total contained 100 PCL chains with five monomers each, resulting in total box sizes of about 2.89 x 2.48 x 20 nm³. To model the interaction in the Al_2_O_3_ crystal, Buckingham parameters^[11]^ have been used, whereas PCL and LiTFSI were modeled by the OPLS-AA^[12]^ and CL&P force fields^[13]^. The interaction between PCL/LiTFSI and Al_2_O_3_ was described by the Universal Force Field (UFF)^[14]^, as recently reported for a similar hybrid electrolyte^[15]^. To implicitly mimic polarization effects, the LiTFSI charges were scaled by a factor of 0.7^[16–18]^, also corresponding to the partial charge of Li^+^ in the Buckingham force field for Al_2_O_3_.

All simulations were carried out using the LAMMPS package .^[19]^ The initial structure was relaxed using with a small timestep of 0.1 fs for 5 ns at 500 K using the Nosé-Hoover thermostat .^[20]^ Once a suitable temperature and relaxed structure was achieved, the system was simulated in the *NpT* ensemble for 50 ns to obtain the equilibrium density, using a time step of 1 fs and the Nosé-Hoover thermostat and Parrinello-Rahman barostat^[21]^ at 500 K and 1 bar pressure. Afterwards, a second equilibration was carried out in the *NVT* ensemble with a time step of 2 fs and a length of 250 ns. Once these equilibrated structures were obtained, the systems were replicated both in *x* and *y* direction (i.e. fourfold increase of the system size) to yield larger but nonetheless equilibrated simulation boxes (roughly 5.78 x 4.96 x 20 nm³), which were simulated for another 45-60 ns of production run. Electrostatic interactions were treated by the using standard Ewald summation technique.

For the analysis of diffusion parallel and perpendicular to the Al_2_O_3_ interface in the main text, it is important to bear in mind that finite-size effects of the diffusion coefficient *parallel* to the interface have been demonstrated for MD simulations of confined liquids due to hydrodynamic effects^[22]^, in addition to potential structural effects. In particular, depending on the size of the simulation box the resulting finite-size correction can be either positive or negative. For the present study, we therefore deliberately chose the ratio of the *x* and *y* dimensions on the one hand and the z dimensions on the other hand such that these spurious hydrodynamic interactions are minimal (i.e. via approximate cancellation of the two terms Eq. 6 in Ref.^[22]^). Furthermore, due to the identical size of the systems with the two grafting densities, any residual finite-size effects would be approximately equal, facilitating their direct comparison. The absence of finite-size anistropy effects of the diffusion constant is further corroborated by the fact that at the large values of $\left| z \right|$ both the parallel and the perpendicular diffusion constant approach each other which otherwise would not be the case (see main text).


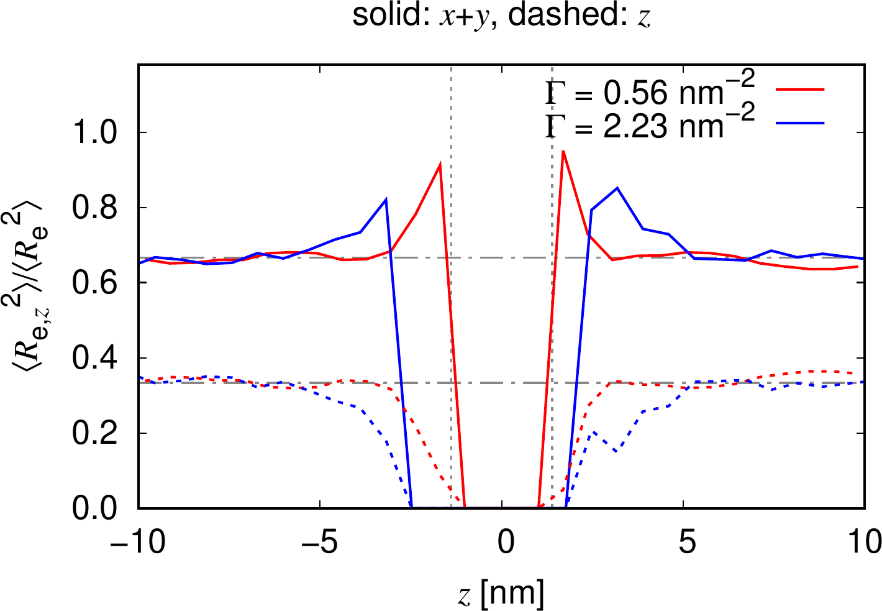


**Figure S2:** Spatial components of the unit end-to-end vector of the free polymer chains in the grafted setup as a function of the *z* axis of the simulation box. The vertical dashed lines represent the boundaries of the Al_2_O_3_ solid, the horizontal dash-dotted lines the values of 1/3 and 2/3 expected for isotropic orientation.

# **Figures on electrochemical and physical investigations**


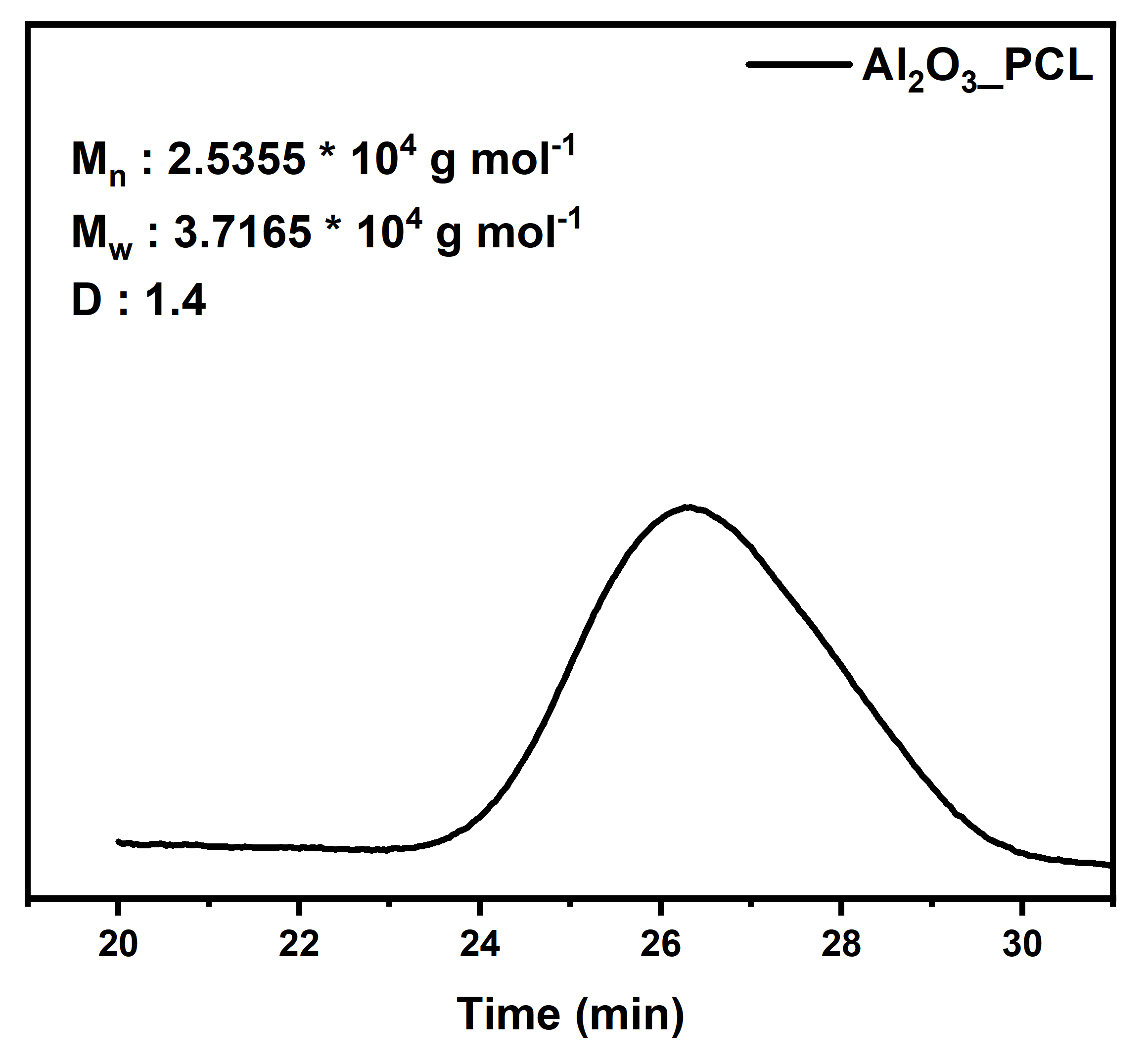


**Figure S3:** GPC spectra of Al_2_O_3__PCL bulk polymer.


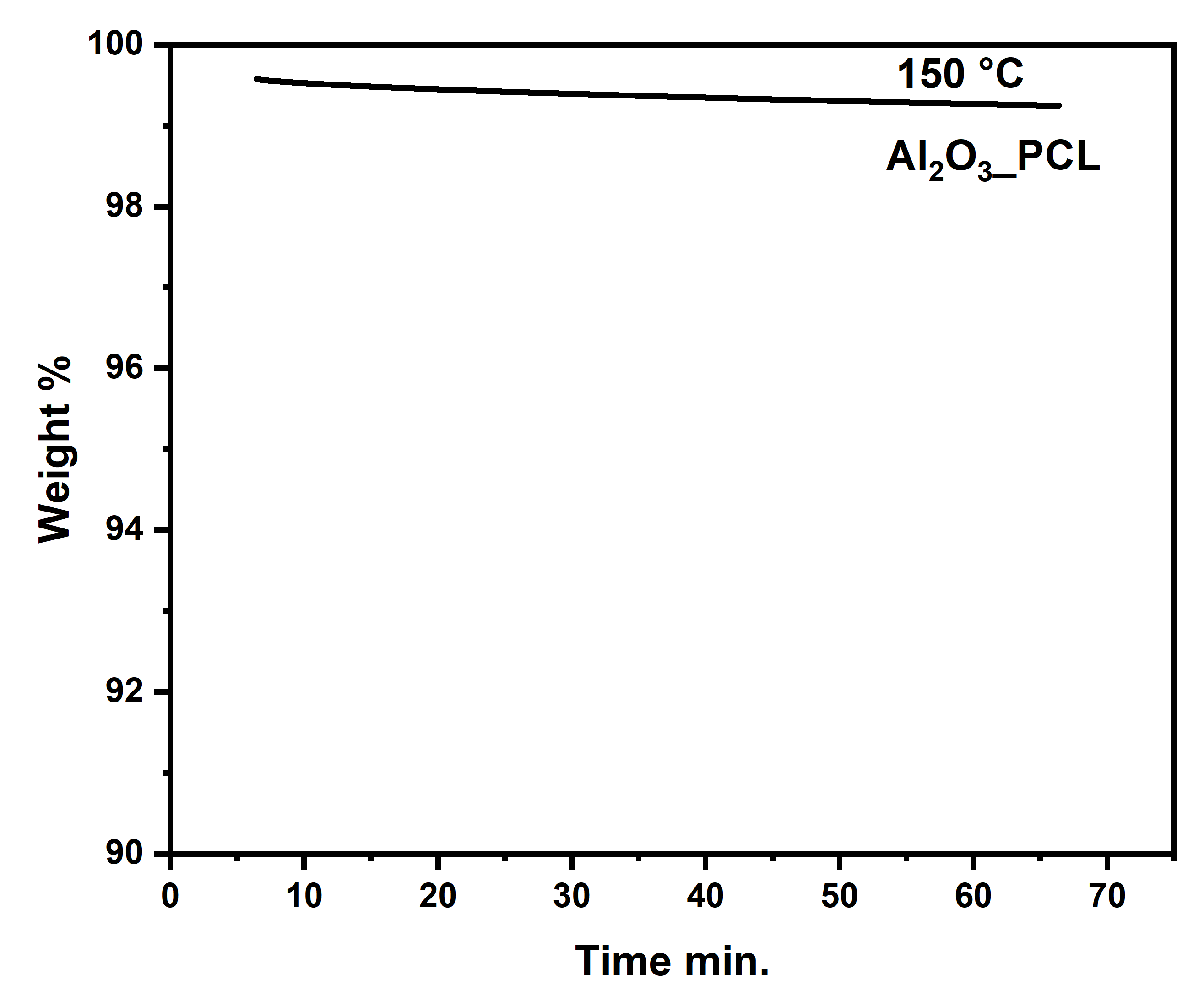


**Figure S4:** Weight loss of Al_2_O_3__PCL at 150 °C for 1h (He flow of 25 mL min^-1^).


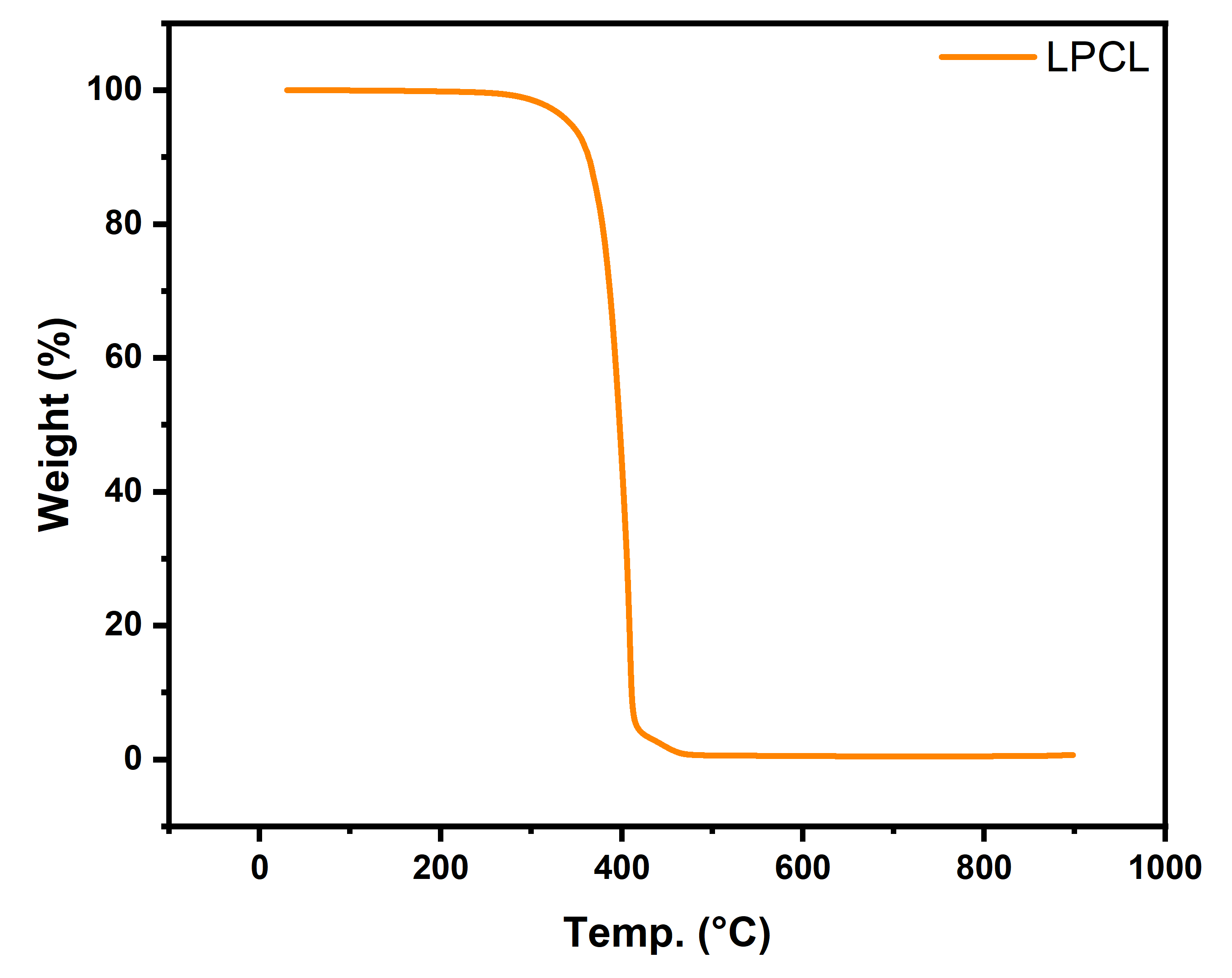


**Figure S5:** TGA curve of linear polycaprolactone.


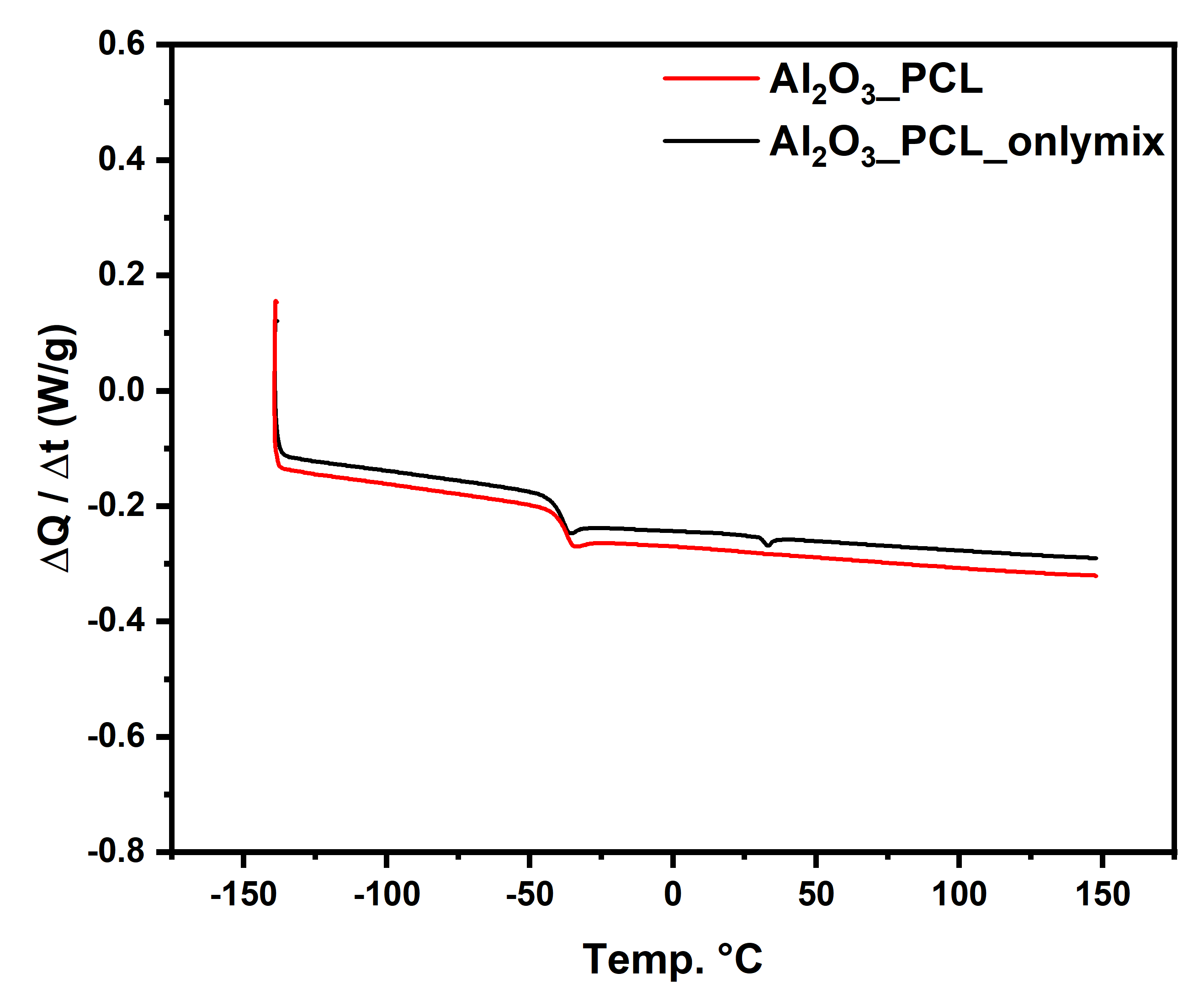


**Figure S6:** DSC curves of Al_2_O_3__PCL and Al_2_O_3__PCL_onlymix electrolyte membranes, second cycle.


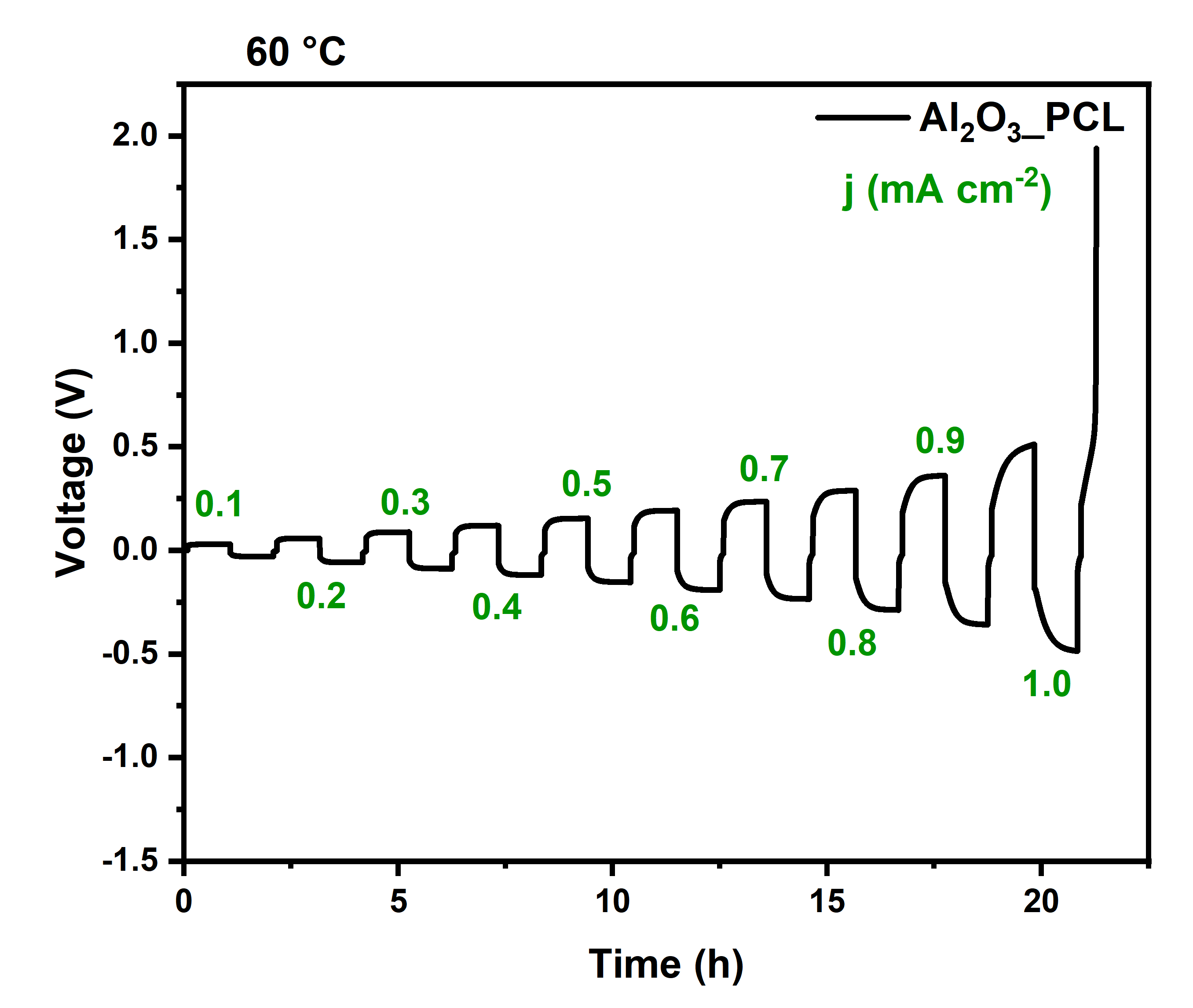


**Figure S7:** C-rate analysis of Al_2_O_3__PCL, ranging from 0.1 to 1.1 mA cm^-2^.


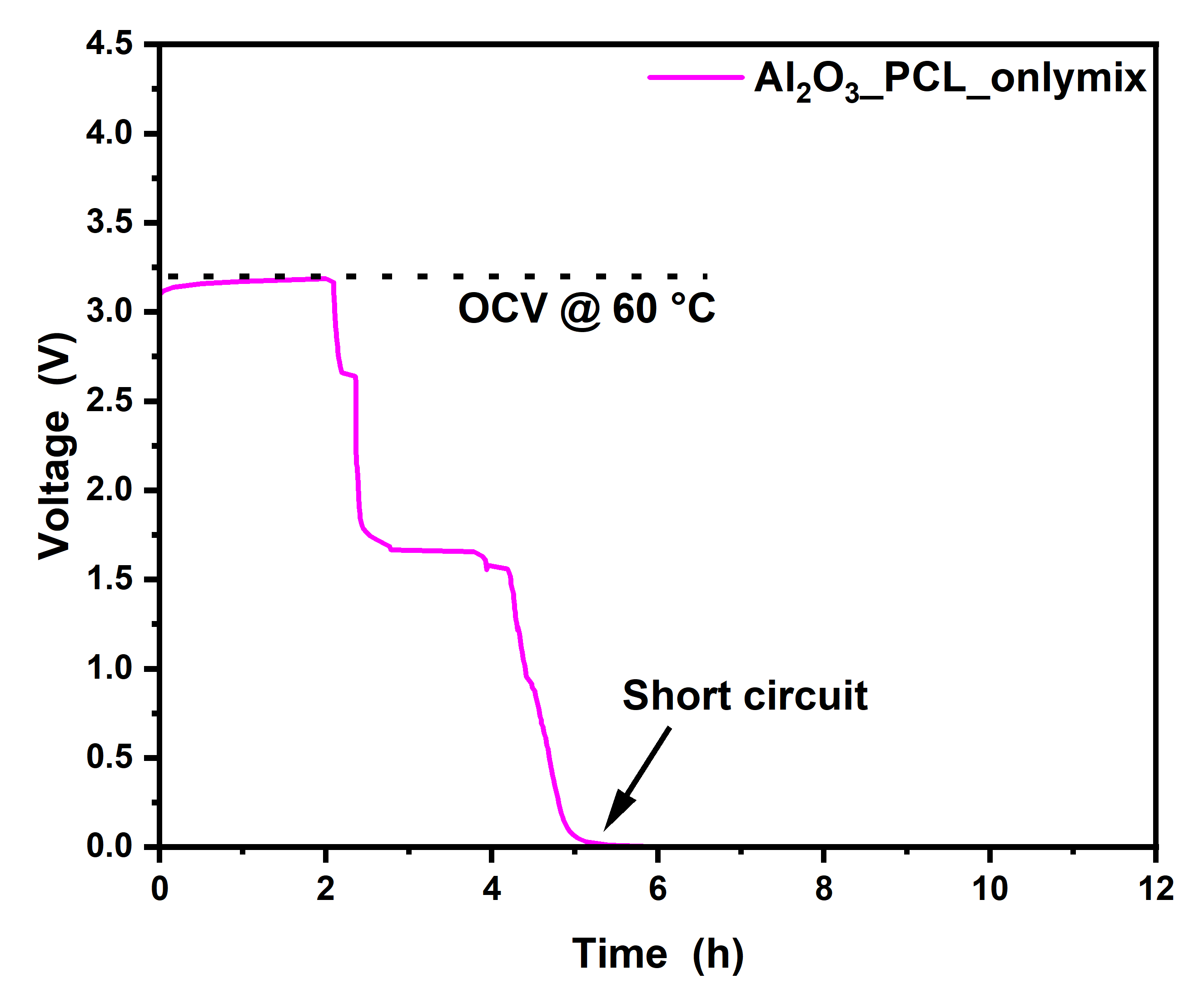


**Figure S8:** Voltage curve of NMC_622_|Al_2_O_3__PCL_onlymix|Li full cell, with a short circuit after 5 h during rest step.

| (a) | 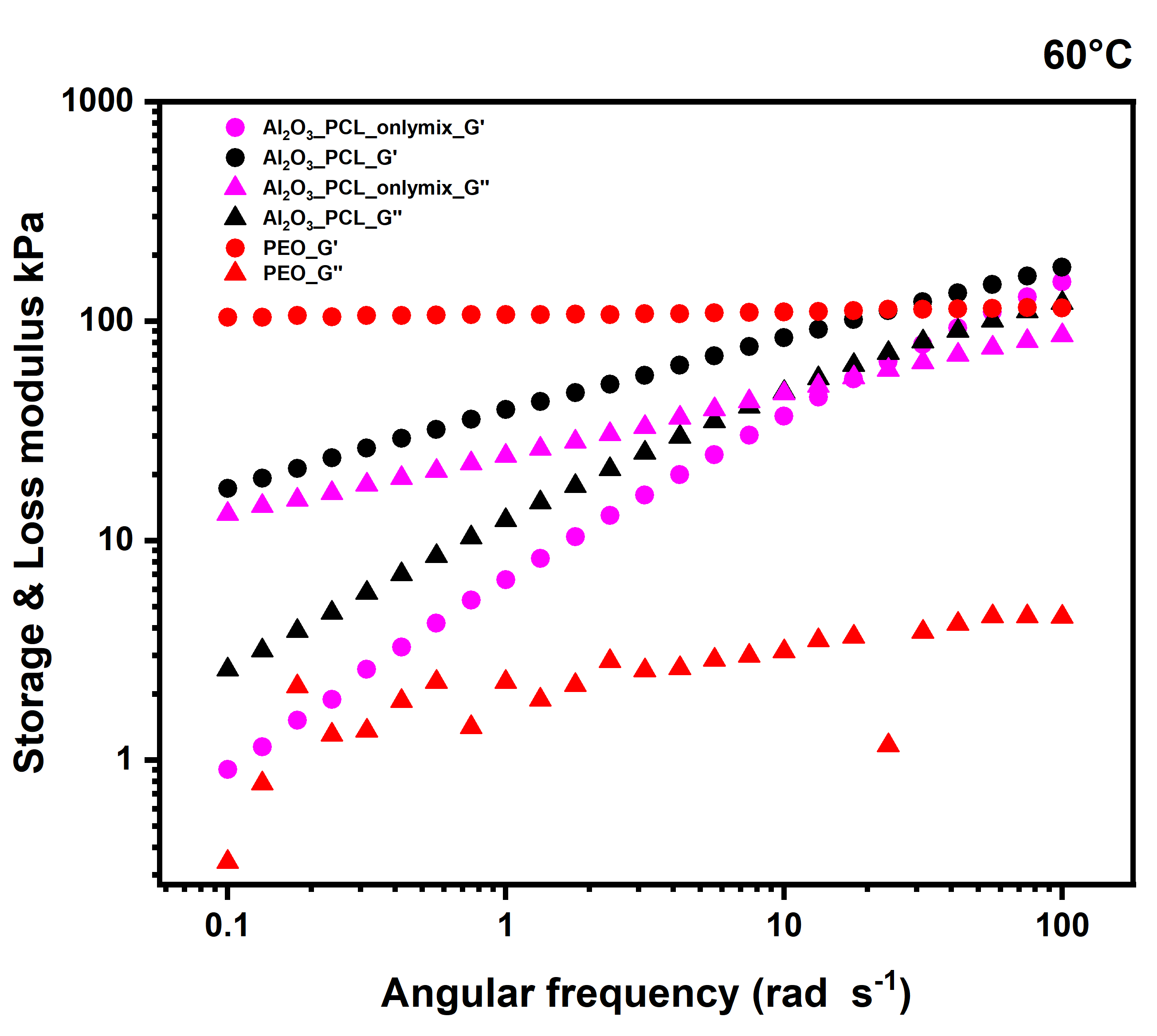 | (b) | 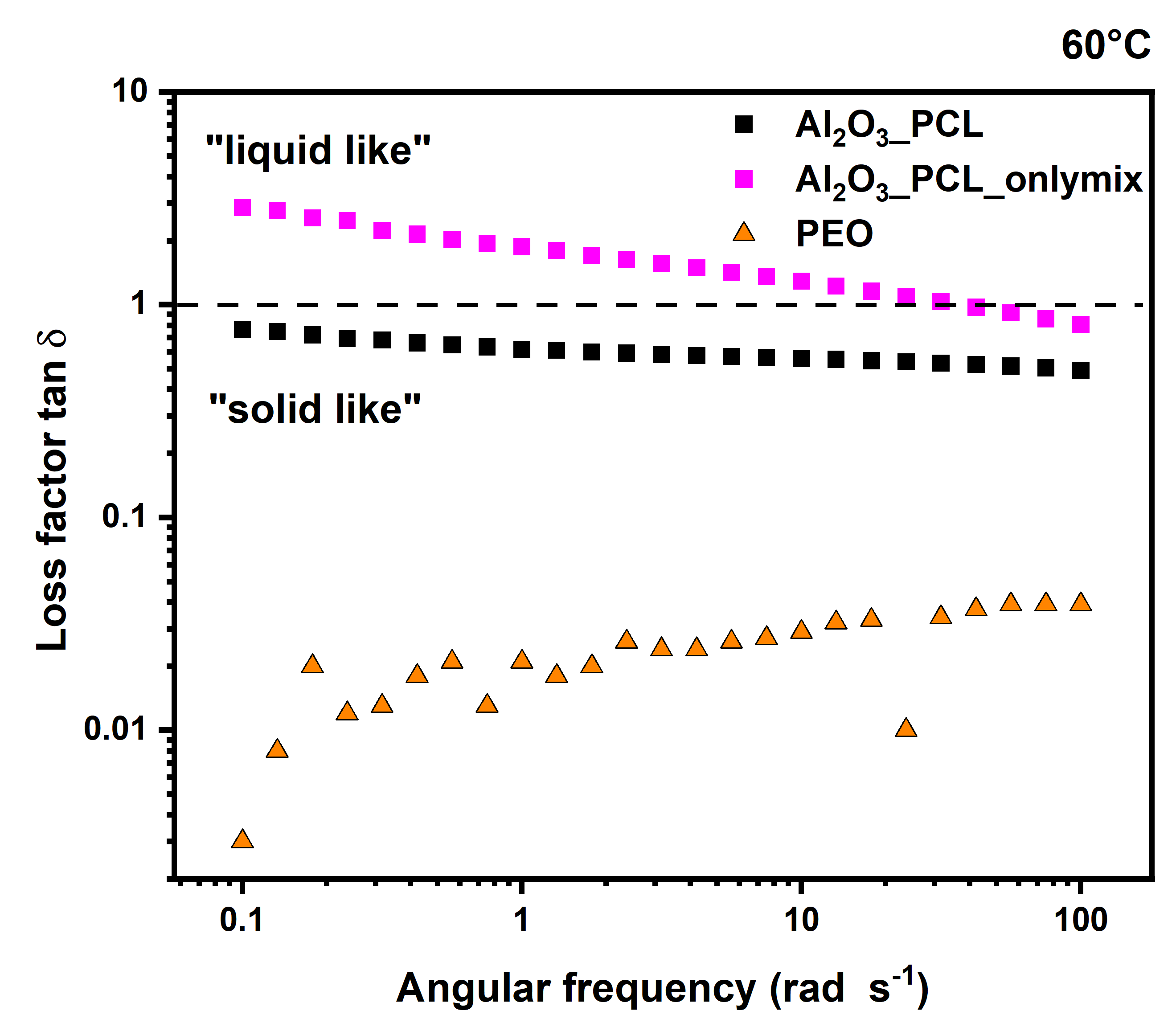 |
| --- | --- | --- | --- |

**Figure S9:** (a) Storage (G´) and loss moduli (G´´) as well as (b) loss factor tan δ versus angular frequency in case of Al_2_O_3__PCL, Al_2_O_3__PCL_onlymix and PEO electrolyte membranes at 60°C.

**Figure S10:** loss factor tan δ versus angular frequency in case of Al_2_O_3__PCL_20wt%, Al_2_O_3__PCL_onlymix, Al_2_O_3__PCL_10wt% and Al_2_O_3__PCL_30wt% electrolyte membranes at 60°C, where Al_2_O_3__PCL_20wt% is referred to as Al_2_O_3__PCL in the manuscript.


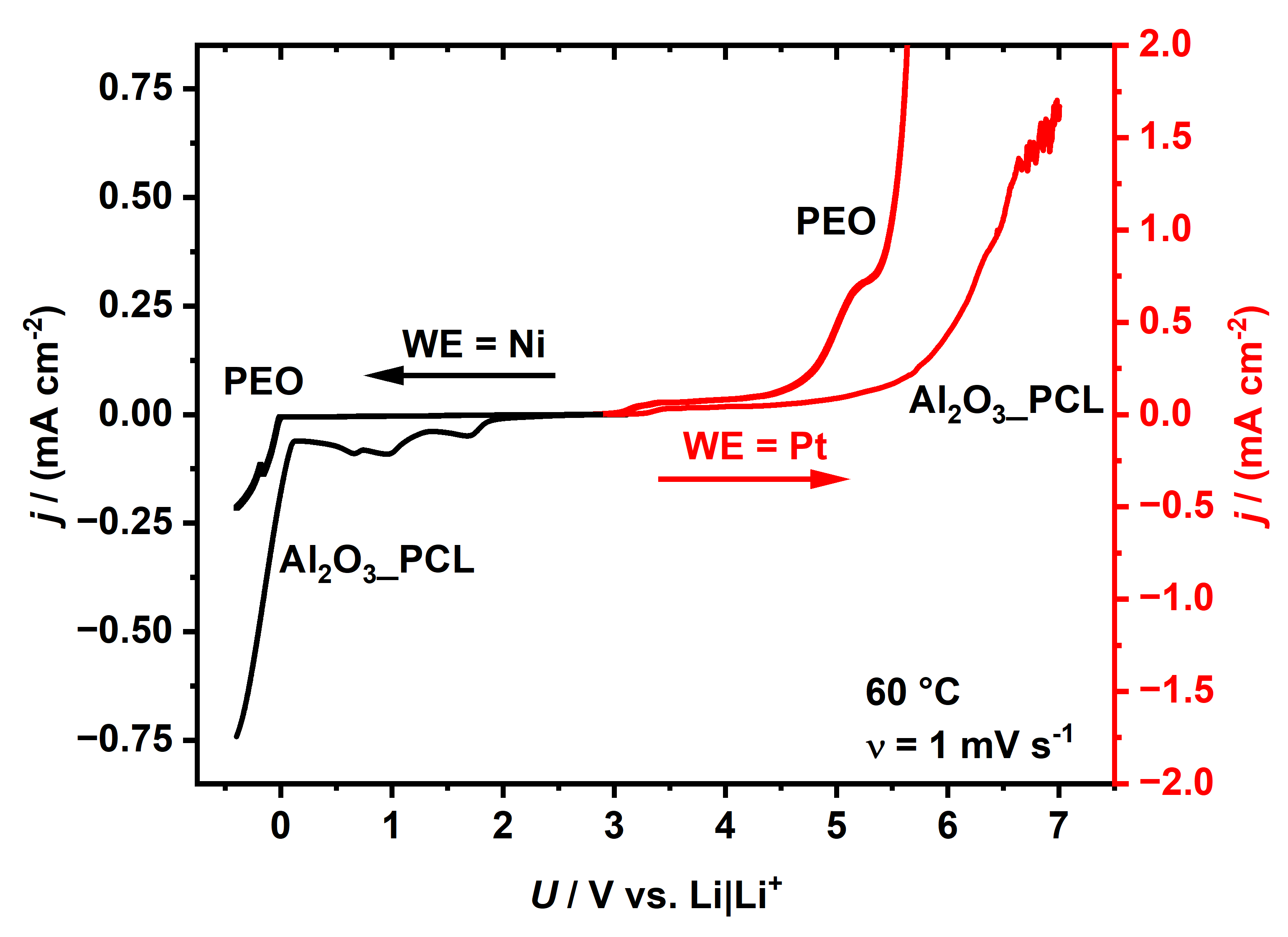


**Figure S11:** LSV curve of Al_2_O_3__PCL and PEO electrolyte membrane at

a scan rate of 1 mVs*^-1^.*

**Figure S12:** Temperature-dependent overall ionic conductivities of LPCL and Al_2_O_3__PCL with 10, 20 and 30 wt% of Al_2_O_3_, where Al_2_O_3__PCL_20wt% is referred to as Al_2_O_3__PCL in the manuscript.

# **Upscaling of Al_2_O_3__PCL with a 2L Reactor**


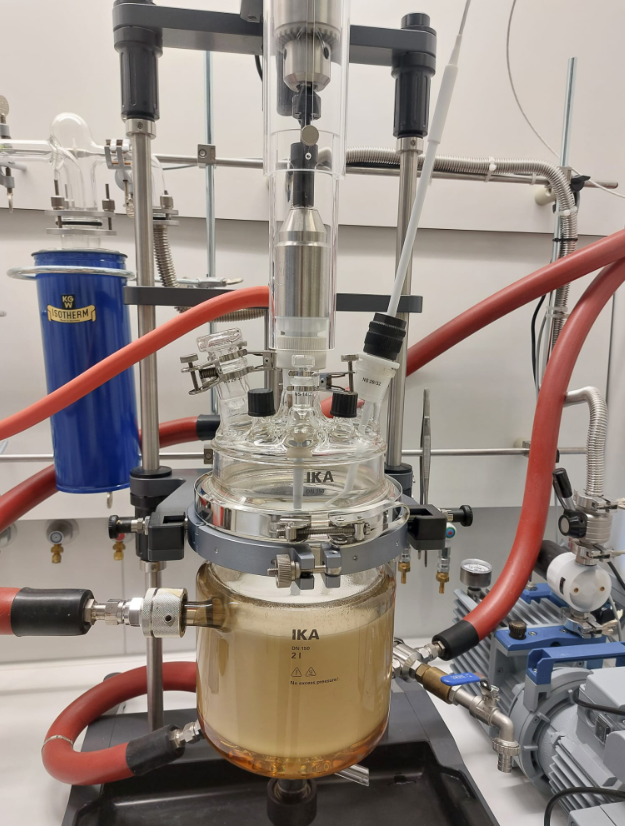


**Figure S13:** 2 L Reactor (IKA) synthesis of Al_2_O_3__PCL

# **Patent research for start-ups working with polymer electrolyte**

**Table S2:** Patent research and comparison of materials and additives used by companies and start-ups in polymer-based cell systems.

| **Blue Current** | **Bolloré** | **Factorial** | **SES** |
| --- | --- | --- | --- |
| „ionically conductive inorganic particles in non-ionically conductive polymer matrix“ | „non-porous polymer matrix and particles of an ionically conductive inorganic material“ | „a polymer, a plasticizer, and an electrolyte salt“. | „high-concentration solvent-in-salt electrolyte“ |
| - Inorganic particles  ≥ 50% of the composition - Lithium oxide argyrodites - Thiophilic metal doped argyrodites - Polymer and sulfide glass - Fluoropolymers - Lithium sulfides | - Inorganic particles ≥ 50% of the composition - Polymer-in-salt electrolyte with inorganic particles - Oxides, lithium complexes, garnet materials - Ceramics or glasses - Block copolymer (PEO + PTFSILi) - Fluoropolymers - Gel electrolytes | - Carbamate-based polymers - More plasticizer than polymer (3:1) - Plasticizer = EC or SN - Inorganic additives: oxides - Acrylate crosslinking - Polylithium acrylate ionomers as artificial SEIs - Ionic liquids and liquid additives - Organic-inorganic hybrids - Polymer + salt + InF3 + solvent | - Polypropylene, polyethylene - Inorganic coatings (lithium silicates, borates, phosphates, etc.) - Cathode + liquid electrolyte Anode + solid coating - Hybrid separators (ceramics/inorganics and polymer) - Polymer composites with inorganics or liquid oligomers - Salt and solvent - Gel polymers |

**SES:** WO2017214276A1; EP3262706B1; US10347904B2; US20220093972A1; US20200280098A1; US20230187650A1; US20200280039A1 ^[23–29]^

**Factorial:** EP3651253B1; US11302960B2; US20190051939A1; US11450884B2; US20230097225A1; US20210020944A1; WO2023014416A1; US20230058958A1; US20230282883A1^[30–38]^

**Bollore:** US11050083B2; US11804619B2; WO2020146948A1; AU2021306495A1^[39–42]^

**Blue Current:** WO2018161047A1; WO2020061354A1; WO2018023079A1; WO2021035243A1; WO2021203060A1^[43–47]^

# **Cost comparison of Al_2_O_3__PCL and PEO electrolytes**

**Table S3:** Cost comparison of Al_2_O_3__PCL and PEO electrolytes.

|  | Al_2_O_3__PCL_(Membrane)_ / € kg^-1^ | PEO_(Membrane)_ / € kg-^1^ |
| --- | --- | --- |
| Polymer | 145.16_(include also_ ${Al}_{2}O_{3}$_)_ | 326.7 |
| LiTFSI | 1230.7 | 1706.4 |
| Benzophenone | 0.9204 | 6.5 |
| **Total** | **1376.78** | **2039.6** |

(Price from Sigma Aldrich & MSE Supplies / Feb. 2024)

# **Table of Electrochemical performance of a variety of mixed and grafted polymer/oxide hybrid electrolytes**

**Table S4:** Electrochemical performance of a variety of mixed and grafted polymer/oxide hybrid electrolytes that have been reported in the literature.

| **Polymer** | **Filler** | **Ratio** | **Li salt** | **Attachment** | **Conductivity** | **Transference number** | **Full cell cycling** | **Current density** | **Active material** | **Capacity** | **Stripping/**  **Plating time** | **Current density** | **Overvoltage** | **Reference** |
| --- | --- | --- | --- | --- | --- | --- | --- | --- | --- | --- | --- | --- | --- | --- |
| PCL | Al_2_O_3_  (5 nm) | 15 wt-% | LiTFSI | only mixed | 1*10^-4^  S cm^-1^  @ 60 °C | \ | 40 | 5  µA cm^-2^ | LFP | 140 mAh g^-1^ | \ | \ | \ | ^[48]^ |
| PEO / liquid | SiO_2_  (25 nm) | 20 wt-% | LiTFSI | grafted | 5*10^-4^  S cm^-1^  @ 60 °C | \ | 100 | 0.2  mA cm^-2^ | NMC | 175 mAh g^-1^ | \ | \ | \ | ^[49]^ |
| PCL | LATP | 40 wt-% | LiClO_4_ | only mixed | 3,64*10^-5^ S cm^-1^  @ 55 °C | 0,58 | 200 | 0,15  mA cm^-2^ | LFP | 137 mAh g^-1^ | 300 h | \ | 160 mV / 60°C | ^[50]^ |
| PEO | Al_2_O_3_  (5 nm) | 32 wt-% | LiTFSI | grafted | 3*10^-4^  S/cm @ 60 °C | 0,08 | 20 | 0.13  mA cm^-2^ | LFP | 150 mAh g^-1^ | \ | \ | \ | ^[51]^ |
| PEO | SiO_2_  (10 nm) | 10 wt-% | LiI | grafted | 5*10^-4^  S cm^-1^  @ 60 °C | \ | \ | \ | \ | \ | \ | \ | \ | ^[52]^ |
| PEO | Al_2_O_3_  (5 nm) | 50 wt-% | 4-methylphenyl)sulfonyl][(trifluoromethyl) sulfonyl)amide | grafted | 6*10^-5^ S/cm @ 60 °C | \ | 130 | \ | LFP | 125 mAh g^-1^ | \ | \ | \ | ^[53]^ |
| PEO | BP | 5 wt-% | LiTFSI | only mixed | 2*10^-4^  S cm^-1^  @ 60 °C | 0,4 | 200 | 0.4  mA cm^-2^ | LFP | 121 mAh g^-1^ | 350 h | 0.7  mA cm^-2^ | 70 mV / 60 °C | ^[54]^ |
| PEO-semi-solid | BP | 0,5 wt-% | LiTFSI | only mixed |  | 0,3 | 100 | 0.8  mA cm^-2^ | LFP | 130 mAh g^-1^ | 550 h | 1  mA cm^-2^ | \ | ^[55]^ |
| PEO | Li_10_SnP_2_S_12_ ("nano") | 1 wt-% | LiTFSI | only mixed | 3*10^-4^  S cm^-1^  @ 60 °C | 0,38 | 40 | \ | Sulfur | 1000 mAh g^-1^ | 600 h | 0.1  mA cm^-2^ | 25 mV / 60 °C | ^[56]^ |
| PVDF / PPC bilayer | LLZO fiber | 30 wt-% | LiFSI | only mixed | 5*10^-4^  S cm^-1^  @ 30 °C | \ | 300 | 0.05  mA cm^-2^ | LFP | 140 mAh g^-1^ | 2700 h | 0.1  mA cm^-2^ | 150 mV / 30 °C | ^[57]^ |
| Polyvinylether / SIC copolymer | \ | \ |  | \ |  | 0,93 | 41 | 0,066 mA cm^.-2^ | LFP | 145 mAh g^-1^ | 1500 h | 0.15  mA cm^-2^ | 300 mV / 30 °C | ^[58]^ |
| PEO | LLZO | 40 wt-% | LiSTFSI | grafted | 1,5*10^-3^  S cm^-1^  @ 60 °C | 0,77 | 120 | \ | LFP | 153 mAh g^-1^ | 1000 h | 0.05  mA cm^-2^ | 23mV / 60 °C | ^[59]^ |
| PEO / plasticizer | 2D Al_2_O_3_ (nanosheets) | 0,5 wt-% | LiTFSI | only mixed | 2,21*10^-3^ S cm^-1^ @60 °C | 0,25 | 50 | 0,425 mA cm^-2^ | LFP | 127 mAh g^-1^ | 400 h | 0.05  mA cm^-2^ | 40 mV / 60 °C | ^[60]^ |
| PCL | LLZO | 40 wt-% | LiTFSI | only mixed | 4,88*10^-5^ S cm^-1^ @60 °C | 0,71 | 600 | 0,39  mA cm^-2^ | LFP | 108 mAh g^-1^ | 700 h | 0.05  mA cm^-2^ | 34 mV / 60 °C | ^[61]^ |
| **PCL** | **Al_2_O_3_**  **(300 nm)** | **20 wt-%** | **LiTFSI** | **grafted** | **5,3*10^-5^**  **S cm^-1^ @60 °C** | **0,65** | **150** | **0,2833 mA cm^-2^** | **NMC** | **150 mAh g^-1^** | **1200 h** | **0,2**  **mA cm^-2^** | **52 mV / 60 °C** | **This work** |

# **References**

[1] P. G. Bruce, M. T. Hardgrave, C. A. Vincent, *Journal of Electroanalytical Chemistry and Interfacial Electrochemistry* **1989**, *271*, 27.

[2] P. G. Bruce, C. A. Vincent, *Journal of Electroanalytical Chemistry and Interfacial Electrochemistry* **1987**, *225*, 1.

[3] W. Zhang, D. A. Weber, H. Weigand, T. Arlt, I. Manke, D. Schröder, R. Koerver, T. Leichtweiss, P. Hartmann, W. G. Zeier, J. Janek, *ACS Appl. Mater. Interfaces* **2017**, *9*, 17835.

[4] A. Latz, J. Zausch, *Journal of Power Sources* **2011**, *196*, 3296.

[5] T. Danner, M. Singh, S. Hein, J. Kaiser, H. Hahn, A. Latz, *Journal of Power Sources* **2016**, *334*, 191.

[6] K. Elbouazzaoui, F. Nkosi, D. Brandell, J. Mindemark, K. Edström, *Electrochimica Acta* **2023**, *462*, 142785.

[7] L. Han, M. L. Lehmann, J. Zhu, T. Liu, Z. Zhou, X. Tang, C.-T. Heish, A. P. Sokolov, P. Cao, X. C. Chen, T. Saito, *Front. Energy Res.* **2020**, *8*, 202.

[8] A. Jain, S. P. Ong, G. Hautier, W. Chen, W. D. Richards, S. Dacek, S. Cholia, D. Gunter, D. Skinner, G. Ceder, K. A. Persson, *APL Materials* **2013**, *1*, 011002.

[9] Agilio Padua, *fftool v1.0.0*, [object Object], **2015**.

[10] L. Martínez, R. Andrade, E. G. Birgin, J. M. Martínez, *J Comput Chem* **2009**, *30*, 2157.

[11] K. Gong, C. E. White, *Cement and Concrete Research* **2021**, *150*, 106588.

[12] W. L. Jorgensen, D. S. Maxwell, J. Tirado-Rives, *J. Am. Chem. Soc.* **1996**, *118*, 11225.

[13] J. N. Canongia Lopes, A. A. H. Pádua, *Theor Chem Acc* **2012**, *131*, 1129.

[14] A. K. Rappe, C. J. Casewit, K. S. Colwell, W. A. Goddard, W. M. Skiff, *J. Am. Chem. Soc.* **1992**, *114*, 10024.

[15] M. Kozdra, D. Brandell, C. M. Araujo, A. Mace, *Phys. Chem. Chem. Phys.* **2024**, *26*, 6216.

[16] I. V. Leontyev, A. A. Stuchebrukhov, *The Journal of Chemical Physics* **2009**, *130*, 085102.

[17] I. Leontyev, A. Stuchebrukhov, *Phys. Chem. Chem. Phys.* **2011**, *13*, 2613.

[18] C. Schröder, *Phys. Chem. Chem. Phys.* **2012**, *14*, 3089.

[19] A. P. Thompson, H. M. Aktulga, R. Berger, D. S. Bolintineanu, W. M. Brown, P. S. Crozier, P. J. In ’T Veld, A. Kohlmeyer, S. G. Moore, T. D. Nguyen, R. Shan, M. J. Stevens, J. Tranchida, C. Trott, S. J. Plimpton, *Computer Physics Communications* **2022**, *271*, 108171.

[20] G. J. Martyna, D. J. Tobias, M. L. Klein, *The Journal of Chemical Physics* **1994**, *101*, 4177.

[21] M. Parrinello, A. Rahman, *Journal of Applied Physics* **1981**, *52*, 7182.

[22] P. Simonnin, B. Noetinger, C. Nieto-Draghi, V. Marry, B. Rotenberg, *J. Chem. Theory Comput.* **2017**, *13*, 2881.

[23] WO2017214276A1 - High energy density, high power density, high capacity, and room temperature capable “anode-free” rechargeable batteries - Google Patents, .

[24] J. Hwang, X. Li, Q. HU, *Electrolte system for high voltage lithium ion battery*, **2018**.

[25] M. Cho, Q. Hu, *Multi-layer polymer coated Li anode for high density Li metal battery*, **2019**.

[26] T. Hakari, A. Tiruvannamalai, L. Zou, M. King, H. Gan, Q. Hu, *Localized High-Salt-Concentration Electrolytes Containing Longer-Sidechain Glyme-Based Solvents and Fluorinated Diluents, and Uses Thereof*, **2022**.

[27] R. P. Singh, S. Nageswaran, Q. Hu, *Free-Solvent-Free Lithium Sulfonamide Salt Compositions That Are Liquid at Room Temperature, and Uses Thereof In Lithium Ion Battery*, **2020**.

[28] Y. Liang, Y. Son, *Functionalized Separators for Electrochemical Cells, Electrochemical Cells Made Therewith, and Methods of Making Each of the Same*, **2023**.

[29] S. Nageswaran, M. King, R. Graves, Q. Hu, *Separators Including Thermally Activated Ionic-Flow-Control Layers, and Electrochemical Devices Incorporating Same*, **2020**.

[30] P. Huang, G. Fu, J. Du, D. REN, *Polymer solid electrolyte, method of making the same, and electrochemical cell*, **2024**.

[31] P. Huang, G. Fu, J. Du, D. Ren, *Polymer solid electrolytes, methods of making, and electrochemical cells comprising the same*, **2022**.

[32] P. Huang, M. Li, Y. Shen, Y. Yu, *Poly(lithium acrylate) and other materials for membranes and other applications*, **2019**.

[33] L. Shen, P. Huang, D. Ren, *Electrolyte, anode-free rechargeable battery, method of forming anode-free rechargeable battery, battery, and method of forming battery*, **2022**.

[34] J. Du, L. Shen, Y. TU, K. Mellott, D. Ren, *Composition, article, method of forming article, anode-free rechargeable battery and forming method thereof, and battery*, **2023**.

[35] Y. TU, G. Fu, J. PARK, D. Ren, *Electrodes for lithium-ion batteries and other applications*, **2021**.

[36] J. Du, D. REN, Y. HUANG, *Electrolyte comprising crosslinked polymer with disordered network*, **2023**.

[37] J. Du, D. Ren, *Article and method of making article*, **2023**.

[38] Y. Huang, J. Du, D. Ren, *Electrochemical Cell Having Electrolyte With Polymer Localized on Electrode Surface*, **2023**.

[39] R. Bouchet, M. Deschamps, *Lithium metal polymer battery having a high energy density*, **2021**.

[40] P. Bernardo, V. Bodenez, M. Deschamps, M. Dru, M. LECUYER, *Solid polymer electrolyte including solvating polymer, lithium salt, and PVdF-HFP copolymer and battery including same*, **2023**.

[41] A. Vallée, P. Leblanc, F. COTTON, B. Guillerm, *Hybrid solid electrolyte for all-solid-state battery*, **2020**.

[42] M. Deschamps, M. LECUYER, J. SZYMCZAK, *Hybrid separating membrane for a battery*, **2023**.

[43] J. BURDYNSKA, A. Teran, B. Rupert, E. Nasybulin, *Polymerized in-situ hybrid solid ion-conductive compositions*, **2018**.

[44] B. Rupert, *Lithium oxide argyrodites*, **2020**.

[45] A. Teran, J. BURDYNSKA, B. Rupert, E. Nasybulin, S. VENUGOPAL, S. K. UPPAL, *Compliant solid-state ionically conductive composite materials and method for making same*, **2018**.

[46] B. Rupert, *Argyrodites doped with thiophilic metals*, **2021**.

[47] I. VILLALUENGA, J. BURDYNSKA, *Byproduct free methods for solid hybrid electrolyte*, **2021**.

[48] T. Eriksson, J. Mindemark, M. Yue, D. Brandell, *Electrochimica Acta* **2019**, *300*, 489.

[49] S. Choudhury, S. Stalin, Y. Deng, L. A. Archer, *Chem. Mater.* **2018**, *30*, 5996.

[50] Y. Li, M. Liu, S. Duan, Z. Liu, S. Hou, X. Tian, G. Cao, H. Jin, *ACS Appl. Energy Mater.* **2021**, *4*, 2318.

[51] E. Fedeli, O. Garcia-Calvo, A. Gutiérrez-Pardo, T. Thieu, I. Combarro, R. Paris, J. Nicolas, H.-J. Grande, I. Urdampilleta, A. Kvasha, *Solid State Ionics* **2023**, *392*, 116148.

[52] Z. Jia, W. Yuan, H. Zhao, H. Hu, G. L. Baker, *RSC Adv.* **2014**, *4*, 41087.

[53] N. Lago, O. Garcia‐Calvo, J. M. Lopez del Amo, T. Rojo, M. Armand, *ChemSusChem* **2015**, *8*, 3039.

[54] N. Wu, Y. Li, A. Dolocan, W. Li, H. Xu, B. Xu, N. S. Grundish, Z. Cui, H. Jin, J. B. Goodenough, *Adv Funct Materials* **2020**, *30*, 2000831.

[55] R. Rojaee, S. Cavallo, S. Mogurampelly, B. K. Wheatle, V. Yurkiv, R. Deivanayagam, T. Foroozan, M. G. Rasul, S. Sharifi‐Asl, A. H. Phakatkar, M. Cheng, S. Son, Y. Pan, F. Mashayek, V. Ganesan, R. Shahbazian‐Yassar, *Adv Funct Materials* **2020**, *30*, 1910749.

[56] X. Li, D. Wang, H. Wang, H. Yan, Z. Gong, Y. Yang, *ACS Appl. Mater. Interfaces* **2019**, *11*, 22745.

[57] Y. Lu, X. Zhang, C. Xue, C. Xin, M. Li, C. Nan, Y. Shen, *Materials Today Energy* **2020**, *18*, 100522.

[58] S. Han, P. Wen, H. Wang, Y. Zhou, Y. Gu, L. Zhang, Y. Shao-Horn, X. Lin, M. Chen, *Nat. Mater.* **2023**, *22*, 1515.

[59] M. Liu, X. Guan, H. Liu, X. Ma, Q. Wu, S. Ge, H. Zhang, J. Xu, *Chemical Engineering Journal* **2022**, *445*, 136436.

[60] J. Song, Y. Xu, Y. Zhou, P. Wang, H. Feng, J. Yang, F. Zhuge, Q. Tan, *Electrochimica Acta* **2023**, *437*, 141504.

[61] A. Wang, D. Pei, Z. Liu, S. Huang, G. Cao, H. Jin, S. Hou, *ACS Appl. Energy Mater.* **2023**, *6*, 8221.
